# Zika Virus Antagonizes Type I Interferon Responses during Infection of Human Dendritic Cells

**DOI:** 10.1371/journal.ppat.1006164

**Published:** 2017-02-02

**Authors:** James R. Bowen, Kendra M. Quicke, Mohan S. Maddur, Justin T. O’Neal, Circe E. McDonald, Nadia B. Fedorova, Vinita Puri, Reed S. Shabman, Bali Pulendran, Mehul S. Suthar

**Affiliations:** 1 Department of Pediatrics, Division of Infectious Diseases, Emory University School of Medicine, Atlanta, Georgia, United States of America; 2 Emory Vaccine Center, Yerkes National Primate Research Center, Atlanta, Georgia, United States of America; 3 Department of Pathology and Laboratory Medicine, Emory University School of Medicine, Atlanta, Georgia, United States of America; 4 J. Craig Venter Institute, Rockville, Maryland, United States of America; NIH, UNITED STATES

## Abstract

Zika virus (ZIKV) is an emerging mosquito-borne flavivirus that is causally linked to severe neonatal birth defects, including microcephaly, and is associated with Guillain-Barre syndrome in adults. Dendritic cells (DCs) are an important cell type during infection by multiple mosquito-borne flaviviruses, including dengue virus, West Nile virus, Japanese encephalitis virus, and yellow fever virus. Despite this, the interplay between ZIKV and DCs remains poorly defined. Here, we found human DCs supported productive infection by a contemporary Puerto Rican isolate with considerable variability in viral replication, but not viral binding, between DCs from different donors. Historic isolates from Africa and Asia also infected DCs with distinct viral replication kinetics between strains. African lineage viruses displayed more rapid replication kinetics and infection magnitude as compared to Asian lineage viruses, and uniquely induced cell death. Infection of DCs with both contemporary and historic ZIKV isolates led to minimal up-regulation of T cell co-stimulatory and MHC molecules, along with limited secretion of inflammatory cytokines. Inhibition of type I interferon (IFN) protein translation was observed during ZIKV infection, despite strong induction at the RNA transcript level and up-regulation of other host antiviral proteins. Treatment of human DCs with RIG-I agonist potently restricted ZIKV replication, while type I IFN had only modest effects. Mechanistically, we found all strains of ZIKV antagonized type I IFN-mediated phosphorylation of STAT1 and STAT2. Combined, our findings show that ZIKV subverts DC immunogenicity during infection, in part through evasion of type I IFN responses, but that the RLR signaling pathway is still capable of inducing an antiviral state, and therefore may serve as an antiviral therapeutic target.

## Author Summary

Zika virus (ZIKV) is an emerging mosquito-borne flavivirus that, upon congenital infection, can cause severe neonatal birth defects. To better understand the early innate immune response to ZIKV, we compared infection of human dendritic cells (DCs) between a contemporary Puerto Rican isolate and historic isolates from Africa and Asia. Human DCs supported productive replication following infection with the contemporary strain and exhibited donor variability in viral replication, but not viral binding. While contemporary and historic Asian lineage viruses replicated similarly, the African strains displayed more rapid replication kinetics with higher infection magnitude and uniquely induced cell death. Minimal DC activation and antagonism of type I interferon (IFN) translation was observed during ZIKV infection, despite strong induction of *IFNB1* transcription and translation of other antiviral effector proteins. Treatment with a RIG-I agonist potently blocked ZIKV replication in human DCs, while type I IFN treatment was significantly less effective. Mechanistically, all ZIKV strains inhibited type I IFN receptor signaling through blockade of STAT1 and STAT2 phosphorylation. Altogether, we found that while ZIKV efficiently evades type I IFN responses during infection of human DCs, RIG-I signaling remains capable of inducing a strong antiviral state.

## Introduction

Zika virus (ZIKV) is an emerging mosquito-borne flavivirus that is causally linked to severe neonatal birth defects upon congenital infection, including microcephaly and spontaneous abortion [1–5], and is associated with Guillain-Barre syndrome [6] and severe thrombocytopenia [7] in adults. ZIKV was first isolated in Uganda in 1947 from a *Rhesus macaque* [8] and later isolated from the mosquito *Aedes africanus* [9]. Phylogenetic analysis has identified three ZIKV lineages, the East African, West African and Asian genotypes, and suggests initial emergence from East Africa and subsequent spread to other regions [10]. Humoral immunity generated against one genotype of ZIKV provides cross-protection against heterologous strains, suggesting the existence of a single ZIKV serotype [11].

For decades, ZIKV remained in Africa and Asia where it sparked local epidemics characterized by mild, self-limiting disease in humans. In recent years, Asian lineage viruses have emerged as a global public health threat with widespread epidemics in Micronesia (2007), the Pacific Islands (2013–2014), and the ongoing outbreak in the Americas (2015–2016), where over 35 countries have reported local transmission [12]. In December of 2015, local transmission of ZIKV was first confirmed in Puerto Rico, where an ongoing and widespread outbreak has caused over 29,345 confirmed cases as of October 20^th^, 2016 [13,14]. Of most concern, local mosquito-borne transmission of ZIKV has been reported in both Texas and Florida and has resulted in a sporadic, yet troubling increase in the number of confirmed cases [15]. Recent human cases and studies in mice have highlighted the role of sexual transmission in spreading ZIKV [16–19], and concerns of transmission through blood transfusions [20] has led to the Federal Drug Administration to advise screening of all blood and blood products for ZIKV. This growing public health crisis underpins the need to better understand viral replication dynamics and the induction of protective immune responses during ZIKV infection.

Dendritic cells (DCs) are critical immune sentinel cells, bridging pathogen detection to activation of innate and adaptive antiviral immunity. Recent studies have found that multiple subsets of murine DCs in the skin and draining lymph nodes [21], as well as human Langerhans cells, dermal DCs, and monocyte derived-DCs are important cells of dengue virus replication [22]. Moreover, a selective loss of type I IFN signaling in DCs ablates host restriction of West Nile virus (WNV), resulting in lethality in a murine infection model [23]. Tick-borne encephalitis virus also interferes with DC maturation through degradation of IRF-1 [24], while Japanese encephalitis virus impairs CD8 T cell immunity through depletion of cross-presenting CD8α+ DCs and impaired up-regulation of MHC class II and the T cell co-stimulatory molecule CD40 [25]. Despite these studies with closely related flaviviruses, the interplay between ZIKV and DCs remains poorly defined.

The retinoic acid-inducible gene I (RIG-I)-like receptor (RLR) and type I IFN signaling axis is essential for inducing an antiviral response during flavivirus infection [26]. The RLRs, which include RIG-I, MDA5, and LGP2, are a family of innate viral RNA sensors that reside in the cytoplasm of nearly every cell of the host [27]. RIG-I and MDA5, signaling through the central adaptor protein mitochondrial antiviral signaling (MAVS), act in concert to restrict flavivirus replication by triggering the production of type I IFN, antiviral effector genes, and pro-inflammatory cytokines [23,26,28–30]. Recent work has shown evolutionarily distinct ZIKV strains antagonize innate immunity by targeting STAT2 for degradation, an essential signal transducer downstream of the type I IFN receptor [31]. However, the contributions of the RLR signaling pathway in restriction of ZIKV replication remains unknown.

In this study, we demonstrate that human DCs are permissive to productive infection by a contemporary Puerto Rican ZIKV. We observed variation in virus replication between individuals, despite similar levels of viral binding to cells. Historic ZIKV isolates from Africa and Asian also infected human DCs, wherein African lineage viruses replicated more rapidly and reached a higher infection magnitude, while also uniquely inducing cell death. During infection with either contemporary or historic ZIKV strains, we observed minimal up-regulation of DC activation markers and pro-inflammatory cytokine secretion. ZIKV infection of human DCs led to significant induction of *IFNB1* at the transcript level, however, we observed impaired translation of type I IFN proteins despite induced protein expression of the RLRs (RIG-I, MDA5, and LGP2), STAT proteins (STAT1 and 2), and antiviral effectors (IFIT1, IFIT3, and viperin). Treatment with a highly specific RIG-I agonist, but not type I IFN, strongly restricted ZIKV replication in human DCs. The impaired ability of type I IFN to block infection reflected viral antagonism of type I IFN-mediated phosphorylation of STAT1 and STAT2. Altogether, we show that human DCs have limited immunogenicity following ZIKV infection, in part due to viral antagonism of type I IFN responses.

## Results

### Contemporary Puerto Rican ZIKV isolate productively infects human DCs

**Fig 1:**
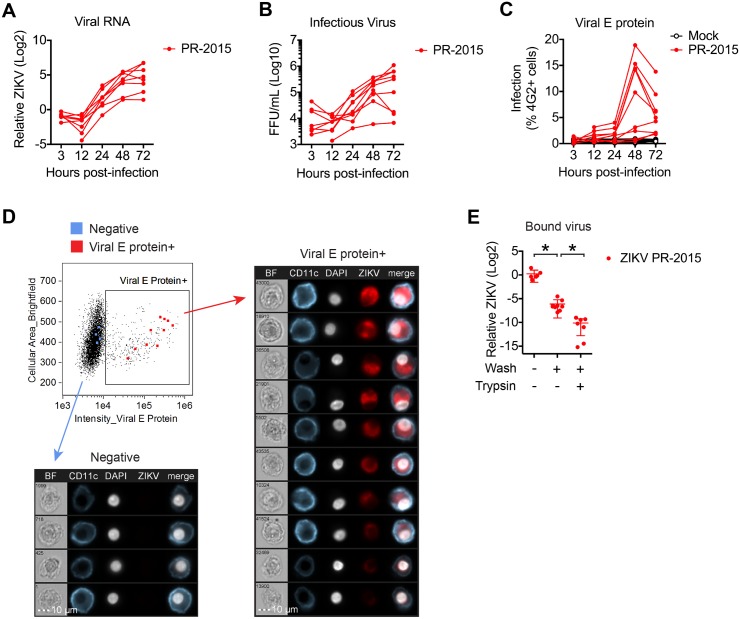
Contemporary Puerto Rican ZIKV isolate productively infects human DCs. moDCs were infected with ZIKV PR-2015 at MOI of 1 and assessed for viral replication at indicated hours post-infection. **(A)** Viral RNA was detected in cell lysates by qRT-PCR for ZIKV E protein RNA. Gene expression is shown as relative expression after normalization to *GAPDH* levels in each respective sample (n = 7 donors). **(B)** Viral titers in supernatants of ZIKV-infected moDCs as determined by focus forming assay (FFA; n = 8 donors). FFU, focus forming units. **(C)** Percent infected cells as assessed by ZIKV E protein staining (4G2-APC antibody) and flow cytometry (n = 9 donors). **(D)** ImageStream analysis of ZIKV-infected moDCs labeled for viral E protein at 48hpi. Images of individual cells highlighted in the flow plot are represented and ordered according to E protein staining intensity. **(E)** moDCs were infected with ZIKV PR-2015 at MOI of 1 for 1hr on ice, washed extensively, and bound virus was quantitated by qRT-PCR for ZIKV RNA. Gene expression is represented as relative expression after normalization to *GAPDH* levels in each respective sample and shown as the mean +/- SD from 6–9 donors. See also S1 Fig.

To understand viral replication in human DCs, we generated monocyte derived-DCs (moDCs) from healthy donors and challenged with PRVABC59, a low passage and sequence-verified ZIKV strain isolated in December of 2015 from the serum of a patient infected while traveling in Puerto Rico (hereafter referred to as “PR-2015”). Genome sequencing and phylogenetic analysis have revealed PR-2015 is closely related to clinical isolates responsible for the 2015–2016 outbreak in Brazil [10,32]. To comprehensively profile PR-2015 replication kinetics in human moDCs, we performed parallel analyses of viral RNA synthesis and release of infectious virus. Viral replication began between 12 and 24 hours post infection (hpi), as evidenced by notable increases in viral RNA synthesis that plateaued between 48 and 72hpi (Fig 1A). No viral RNA was detected in mock-infected cells. The kinetics of viral RNA synthesis corresponded to increased release of infectious virus between 12 and 24hpi with continued log phase growth through 48hpi (Fig 1B). Together, our findings show that human moDCs support productive ZIKV replication with a contemporary Puerto Rican strain.

### Cellular level analysis of Puerto Rican ZIKV replication in human DCs

We next determined how PR-2015 infection at the single cell level impacts viral growth kinetics in the bulk cell population. Infected moDCs were labeled for expression of ZIKV antigen using the pan-flavivirus 4G2 antibody, which recognizes a structural protein found within the virus envelope (E), and percent infection was assessed by flow cytometry. Infected cells were first detected in low numbers at 12hpi (Mock, 0.2–0.9%; PR-2015, 0.2–3.2%), increasing in percentage and staining intensity over the next 36 hours (Fig 1C). When we infected moDCs with ultraviolet (UV)-inactivated virus, we observed no E protein staining above uninfected cells, confirming detection of newly synthesized viral protein (S1A Fig). To confirm antibody staining, we performed ImageStream analysis of PR-2015-infected moDCs. ZIKV E protein was detected within the cytoplasm and did not co-localize with the cell surface marker CD11c (Fig 1D). This staining pattern is consistent with our recent observations of ZIKV E protein staining in placental macrophages, where viral protein localized to perinuclear regions within the cytoplasm and likely within an endoplasmic reticulum-derived network [33,34]. As expected, increases in the percentage of infected cells corresponded to the kinetics of viral RNA synthesis and infectious virus release.

### Variability in Puerto Rican ZIKV infection occurs after viral binding

Notably, moDCs generated from four of the nine donors used in this analysis released lower amounts of infectious virus, and in some cases, synthesized lower amounts of viral RNA (Fig 1A and 1B). When we directly compared infectious virus release and viral E protein staining, the same 4 donors with the lowest amount of infectious virus release at 48 and 72hpi (“low infection”) also had lower percentages of E protein positive cells at 48hpi (0.4–3.1%) as compared to the other 5 “high infection” donors (9.8–18.9%) (S1B Fig). One explanation for variability in viral replication may be differences in viral binding to host receptors on moDCs generated from different donors. To test this, we developed a qRT-PCR-based viral binding assay (S1C Fig) [35,36]. To verify we were measuring bound virus, we compared viral RNA levels with and without washing, as well as after trypsin treatment, which should cleave proteinaceous cellular receptors and remove bound virus from the cell surface. Washing cells significantly reduced the amount of virus detected and trypsin treatment further diminished viral RNA levels, confirming our ability to measure cell-bound virus in the “+Wash, -Trypsin” condition (Fig 1E). In contrast to the differences observed in viral RNA synthesis, viral E protein staining, and infectious virus release, there was minimal difference in the amount of bound virus between different donors. This suggests that the variability in ZIKV infection between donors occurs after viral binding.

### Differential infection of human DCs by evolutionarily distinct ZIKV strains

We next infected moDCs with sequence-verified ZIKV isolates spanning the evolution of the virus since its discovery, including ancestral isolates from East Africa (MR-766, “MR-1947”), West Africa (DakAr 41524, “Dak-1984”), and Asia (P6-740, “P6-1966”) [10,37]. The MR-1947 strain was isolated in 1947 from an infected sentinel *Rhesus macaque*, monkey number 766, in the Ziika forest of Uganda. The Dak-1984 strain was later isolated from an infected *Aedes africanus* mosquito in Senegal in 1984. The P6-1966 strain was isolated in 1966 from an infected *Aedes aegypti* mosquito in Malaysia, and represents the oldest known ancestor of the Asian lineage since divergence from the African lineages. Each viral isolate has different laboratory passage histories, including multiple passages in suckling mouse brains in the case of MR-1947 and P6-1966, which must also be taken into consideration (S1 Table). We independently sequenced each of the four ZIKV strains and performed nucleotide sequence alignments (see S1 Table for genome accessions), finding P6-1966 shares 95.5% of its coding region with PR-2015, while MR-1947 and Dak-1984 only share 88.6% with PR-2015 (S1 Table). This corresponded to 1.1%, 3.2%, and 3.0% differences in amino acids between PR-2015 and P6-1966, MR-1947, and Dak-1984, respectively. Of note, MR-1947 diverged from PR-2015 more notably in the structural (4.4%) than non-structural proteins (2.9%).

**Fig 2:**
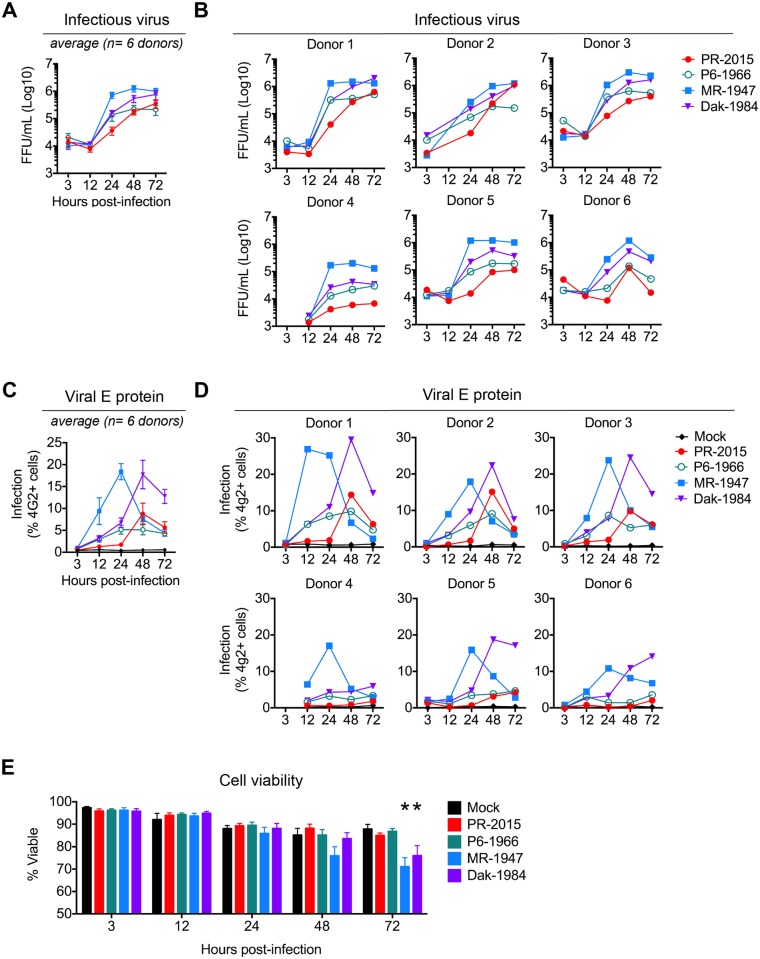
Differential infection of human DCs by evolutionarily distinct ZIKV strains. moDCs were infected with PR-2015, P6-1966, MR-1947, or Dak-1984 at MOI of 1 and assessed for viral replication at the indicated hours post-infection. **(A)** Infectious virus release into the supernatant was determined by FFA. Shown as the mean +/- SEM from 6–9 donors. **(B)** Infectious virus release for 6 of the individual donors summarized in panel A. **(C)** Percent infected cells assessed by ZIKV E protein staining and flow cytometry. Shown as the mean +/- SEM from 6–9 donors. **(D)** Percent infected cells in 6 of the individual donors summarized in panel C. **(E)** Cell viability of infected moDCs assessed by Ghost Red 780 (Tonbo) viability staining and flow cytometry. Shown as the mean +/- SEM from 6–9 donors. Statistical significance (p< 0.05) was determined using a two-way ANOVA with comparisons made to mock-infected cells. See also S1 Table.

Using the same moDCs generated from six of the previous donors (Fig 1), we directly compared infection kinetics of the ancestral strains with that of PR-2015. The infections were performed in parallel with PR-2015 to allow for direct cross-comparison of viral growth between the different viral strains (S2A Fig). MR-1947 exhibited rapid replication kinetics with increased infectious virus release and viral RNA synthesis occurring between 12 and 24hpi (Fig 2A, 2B, and S2B Fig). The percentage of infected cells peaked at 24hpi (Fig 2C and 2D). We next compared MR-1947 replication with Dak-1984, which is closely related to MR-1947 but has undergone less laboratory passaging. Despite reaching a similar infection magnitude, Dak-1984 exhibited slower growth kinetics as compared to MR-1947, with percent infection and release of infectious virus peaking between 48 and 72hpi. The P6-1966 strain replicated with similar kinetics and magnitude to PR-2015 through 48hpi, although we did observe subtle differences in virus infection. In particular, P6-1966 replicated to modestly higher levels at 24hpi than PR-2015. Despite this, P6-1966 replication plateaued more rapidly than PR-2015 and failed to reach a comparable magnitude of infection. These subtle differences may reflect genetic changes between ancestral Asian lineage strains and current circulating strains (S1 Table). Of note, P6-1966 was found to produce smaller viral plaques and foci on Vero cells as compared to the other three strains (S2C Fig). Given recent studies linking ZIKV to cell death of neural progenitor cells, we evaluated cell viability during ZIKV infection of human moDCs [38–40]. While MR-1947 and Dak-1984 induced significant decreases in cell viability by 72hpi, neither of the Asian lineage strains resulted in loss of viability as compared to time-matched, uninfected cells (Fig 2E). Together, our data suggest that evolutionarily distinct ZIKV strains exhibit varying replicative and cell death capacities during infection of human DCs.

### Differential susceptibility of human DCs to ancestral and circulating ZIKV strains

Given our findings that moDCs generated from different donors have differential susceptibilities to PR-2015 infection (Fig 1), we next compared replication between the four strains on a donor-by-donor basis. The MR-1947 strain replicated well within all donors, showing the least amount of variation in viral replication between donors (Fig 2B, 2D and S1B Fig). The Dak-1984 strain also replicated well in most donors, albeit to modestly diminished peak levels in donors with lower levels of PR-2015 replication. In contrast, P6-1966 replicated similarly to PR-2015 for a given donor, likely representing their shared ancestry. Together, these data suggest both viral factors, as found between different strains, as well as non-viral factors, as found between different donors, influence ZIKV replication in human DCs.

### ZIKV infection minimally activates human DCs

**Fig 3:**
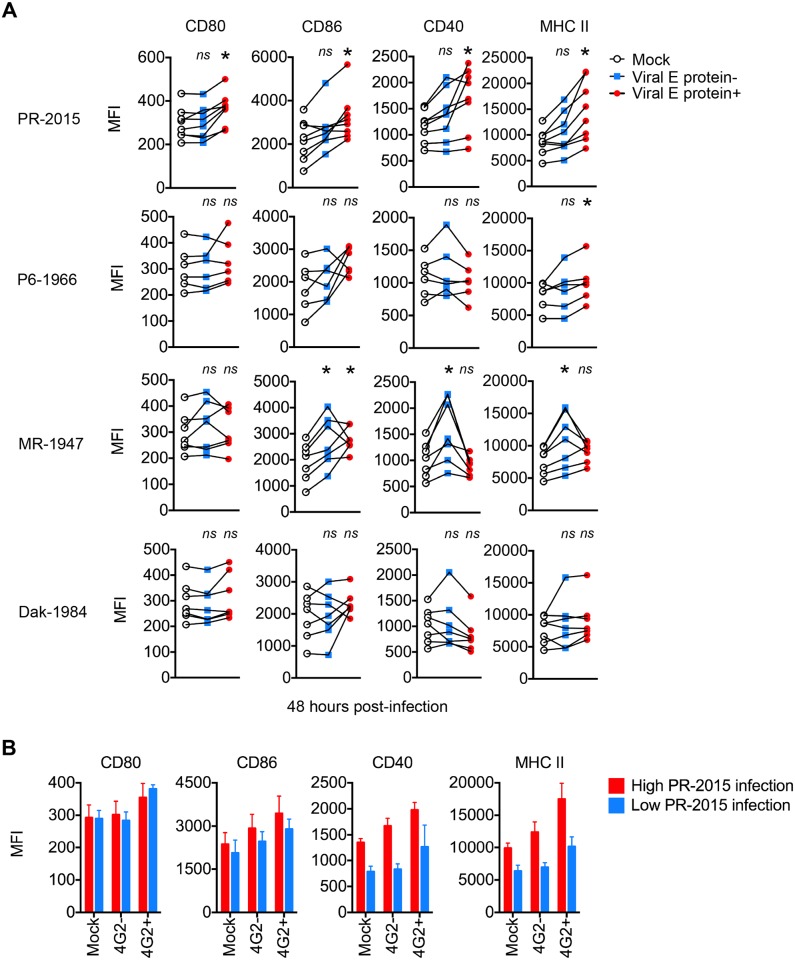
ZIKV infection minimally activates human DCs. **(A)** moDCs were left uninfected (“Mock”) or infected with PR-2015, P6-1966, MR-1947, or Dak-1984 at MOI of 1 (n = 6–8 donors). Cells were collected at 48hpi and labeled for ZIKV E protein and indicated DC activation markers. Cells were categorized as being viral E protein- or viral E protein+ and activation marker surface expression quantitated by flow cytometry. Values are represented as median fluorescence intensity (MFI) for each individual donor with uninfected and ZIKV infected samples from the same donor connected with a line. Statistical significance (p< 0.05) was determined using a Friedman test with comparisons made to donor-paired, uninfected cells. **(B)** moDCs infected with PR-2015 at MOI of 1 were stratified into “low” (n = 3 donors) and “high” (n = 5 donors) infection on the basis of viral E protein staining. MFIs are shown as the mean +/- SD. See also S3 Fig.

A critical function of DCs is the programming of virus-specific T cell responses that are required for clearance of virally infected cells. Engagement of virus-associated molecular patterns increases the surface expression of co-stimulatory and MHC molecules on activated DCs, potently enhancing their ability to prime virus-specific T cell responses [41]. To determine the ability of ZIKV infection to program DCs, we measured cell surface expression of co-stimulatory (CD80, CD86, and CD40) and MHC class II molecules at 48hpi with all four ZIKV strains. We labeled cells with 4G2 antibody and divided infected samples based on viral E protein staining (E protein-, bystander cells and E protein+, infected cells). Following infection with PR-2015, we observed significant but modest activation in E protein+ cells only, while infection of moDCs with P6-1966 or Dak-1984 induced minimal activation (Fig 3A). In comparison, MR-1947 induced modest activation, but primarily within the E protein- cell population. This is in contrast to strong activation induced by RIG-I agonist treatment of moDCs (S3A Fig). Next, we confirmed our findings in more physiologically relevant human antigen presenting cell subsets. Similar to moDCs, *ex vivo* infection of primary monocytes, myeloid DCs, and plasmacytoid DCs from the blood of healthy donors failed to induce up-regulation of co-stimulatory or MHC molecules (S3B–S3D Fig).

We next asked whether the donor variability in viral replication with PR-2015 (Fig 1) corresponded to differences in DC activation during infection. We grouped samples into “low” or “high” infection donors on the basis of viral E protein staining (S1B Fig). We found no differences in the up-regulation of CD80 and CD86 when we stratified by viral replication (Fig 3B). In contrast, both CD40 and MHC class II showed greater up-regulation during infection of moDCs from donors with higher viral replication. This suggests that the induction of DC activation is influenced by the magnitude of viral replication. Altogether, these data show that ZIKV induces minimal DC activation and as a consequence, infected DCs may be compromised in their ability to prime antiviral T cell responses.

### ZIKV does not induce pro-inflammatory cytokine secretion by human DCs

**Fig 4:**
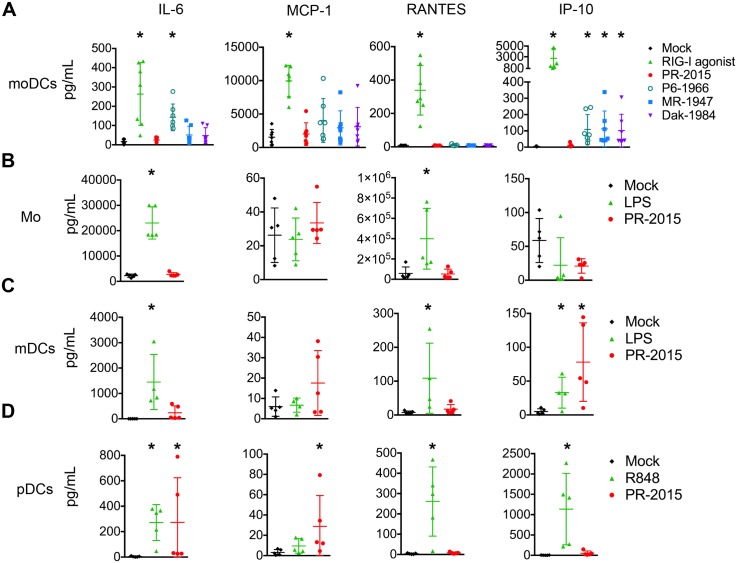
ZIKV infection induces minimal pro-inflammatory cytokine production by DCs. **(A)** moDCs were left untreated (“Mock”), transfected with RIG-I agonist (10ng/1e5 cells), or infected with PR-2015, P6-1966, MR-1947, or Dak-1984 at MOI of 1 (n = 7 donors). Supernatants were collected at 48hpi. **(B, C)** Monocytes (Mo) and myeloid DCs (mDCs) were left untreated (“Mock”), treated with LPS (100 ng/ml), or infected with PR-2015 at MOI of 1 (n = 5 donors). Supernatants were collected at 24hpi. **(D)** Plasmacytoid DCs (pDCs) were left untreated (“Mock”), treated with R848 (1 μg/ml), or infected with PR-2015 at MOI of 1 (n = 5 donors). Supernatants were collected at 24hpi. Cytokine production was assessed using multiplex bead array. Values for each individual donor are shown with the mean +/- SD. Statistical significance (p< 0.05) was determined using a Kruskal-Wallis test with comparisons made to untreated (“Mock”) cells. See also S2, S3, S4, and S5 Tables.

In addition to providing T cell co-stimulation, DCs promote innate and adaptive immunity through the secretion of pro-inflammatory mediators. We next assessed inflammatory cytokine and chemokine release following PR-2015 infection of moDCs. Consistent with minimal increases in surface expression of co-stimulatory molecules, PR-2015 infection failed to induce the secretion of most pro-inflammatory cytokines assayed, despite the ability of RIG-I agonist to induce their secretion (Fig 4A and S2 Table). The ancestral strains also failed to induce substantial cytokine release during infection of human moDCs. Of exception, P6-1966 induced significant IL-6 secretion, and along with MR-1947 and Dak-1984, triggered modest yet significant IP-10 secretion. Finally, to confirm these findings in more physiologically relevant myeloid cell subsets, we stimulated primary monocytes (Fig 4B and S3 Table), myeloid DCs (Fig 4C and S4 Table), and plasmacytoid DCs (Fig 4D and S5 Table) isolated from healthy human blood with PR-2015 to assess cytokine and chemokine secretion. Despite the ability of LPS (monocytes and myeloid DCs) or R848 (plasmacytoid DCs) to induce cytokine production, infection with ZIKV did not promote notable pro-inflammatory cytokine secretion. Together, our data suggests that human antigen-presenting cells exposed to ZIKV are compromised in their ability to promote inflammatory responses.

### Human DCs infected with ZIKV secrete minimal type I and III IFNs

During viral infection, early innate immune signaling triggers the production of type I and III IFNs and antiviral effector molecules that block viral replication [42]. In particular, RLR and type I IFN signaling are essential for host restriction of flavivirus replication and ultimate control of infection [23,26,43]. Specific to ZIKV, mice with intact type I IFN responses support only limited and low level viral replication, while genetic ablation or antibody blockade of type I IFN signaling shifts the balance towards sustained, high level ZIKV replication and pathology, including neuroinvasive disease [44–46]. Moreover, mice deficient in their ability to produce type I IFN are similarly compromised in their ability to restrict viral replication [16,46].

**Fig 5:**
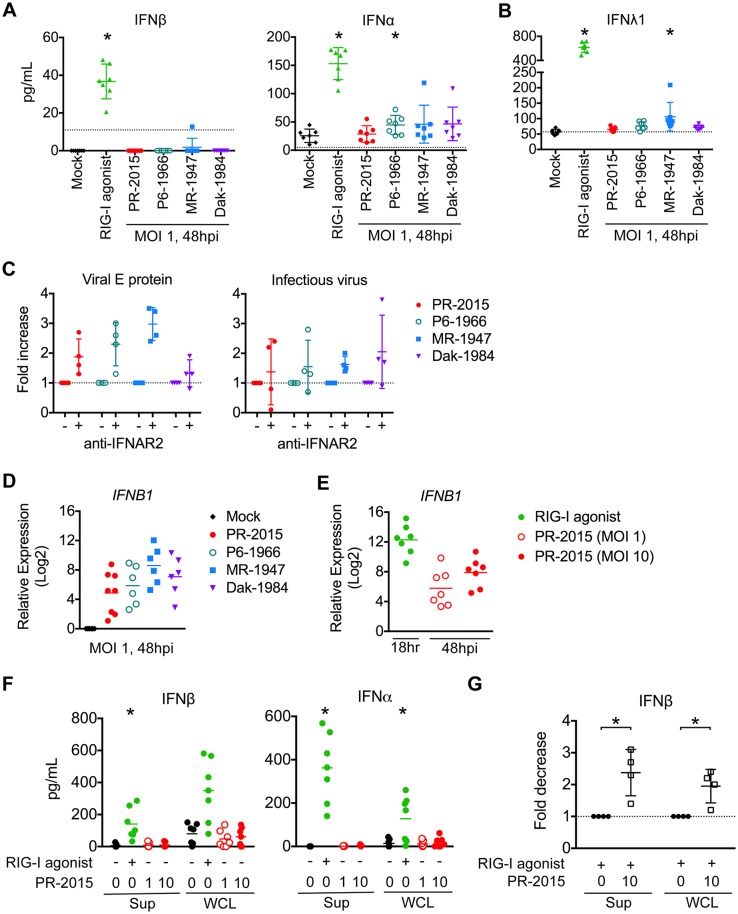
ZIKV infection induces type I IFN transcription but inhibits translation. moDCs were left untreated (“Mock”), treated with RIG-I agonist (10ng/1e5 cells), or infected with PR-2015, P6-1966, MR-1947, or Dak-1984 at MOI of 1. Supernatants were collected 24hrs (RIG-I agonist treatment) or 48hrs (ZIKV infection) later and IFNβ and IFNα **(A)** or IFNλ1 **(B)** production was assessed via multiplex bead array. Values for each individual donor are shown with the mean +/- SD (n = 7 donors). Statistical significance (p< 0.05) was determined using a Friedman test with comparisons made to donor-paired, mock-infected cells. A dashed line indicates the assay limit of detection. **(C)** moDCs were infected with ZIKV at MOI of 1 in the presence of anti-IFNAR2 blocking antibody. Cells were collected at 48hpi and labeled for ZIKV E protein, while release of infectious virus into the supernatants was determined by FFA. Values for each individual donor are shown with the mean +/- SD (n = 4 donors). A dashed indicates no change relative to infection in the absence of anti-IFNAR2 blocking antibody. **(D)** RNA was harvested from cells treated the same as for cytokine analysis and *IFNB1* mRNA expression was determined by qRT-PCR. Gene expression was normalized to *GAPDH* transcript levels in each respective sample and represented as the log_2_ normalized fold increase above donor- and time point-matched untreated cells. Values for each individual donor are shown with the mean (n = 6–8 donors). **(E)** moDCs were treated with RIG-I agonist (10ng/1e5 cells, 18hrs) or infected with ZIKV PR-2015 (MOI 1 and 10, 48hrs) and analyzed for *IFNB1* mRNA expression. Values for each individual donor are shown with the mean (n = 7 donors) **(F)** IFNβ and IFNα were measured in the supernatant (“Sup”) and whole cell lysate (“WCL”) of moDCs treated the same as in E. Values for each individual donor are shown with the mean (n = 7 donors). Statistical significance (p< 0.05) was determined using a Friedman test with comparisons made to donor-paired, mock-infected cells. **(G)** Uninfected or ZIKV PR-2015-infected moDCs (MOI 10, 48hpi) were treated with RIG-I agonist (10ng/1e5 cells, 18hrs) and IFNβ and IFNα were measured as in F. The data is shown as the fold-decrease from RIG-I agonist treatment alone with significance (P<0.05) determined using a Mann Whitney Test (n = 4 donors). Error bars represent the mean +/- SD. See also S4 Fig.

To determine the potential of human DCs to trigger type I IFN responses during ZIKV infection, we measured the secretion of type I IFN proteins into the supernatant by infected populations of moDCs at 48hpi. Surprisingly, all four ZIKV strains failed to induce detectable IFNβ secretion and induced only minimal secretion of IFNα (Fig 5A). Given this intriguing finding and the recently appreciated role of type III IFNs in antiviral immunity, we next measured the secretion of IFNλ1 [47]. Similar to type I IFNs, ZIKV infection induced minimal secretion of type III IFN protein (Fig 5B). Treatment of the same donor cells with RIG-I agonist induced significant secretion of all three molecules, confirming these cells are capable of producing type I and type III IFNs. Similar to moDCs, pDCs produced low amounts of IFNα following ZIKV infection (S5 Table).

Next, as a complementary measurement of type I IFN secretion, we infected moDCs with ZIKV in the presence of an anti-IFNAR2 blocking antibody. Blockade of type I IFN signaling enhanced ZIKV infection modestly across all four ZIKV strains, resulting in only a 2–3 fold increase in the percentage of virally infected cells (Fig 5C). Despite this increase in the percentage of infected cells, we observed minimal differences in the release of infectious virus in the presence of anti-IFNAR2 blocking antibody. Combined, these findings suggest that human DCs secrete type I IFN at near undetectable levels during ZIKV infection.

### ZIKV infection of human DCs induces type I IFN transcription, but not translation

Given that multiple pathogenic human viruses have involved mechanisms to interfere with type I IFN transcription [48–51], we next assessed the levels of *IFNB1* transcripts in ZIKV-infected moDCs. Despite near undetectable protein secretion, all four ZIKV strains induced notable *IFNB1* gene transcription at 48hpi, with MR-1947 showing the highest induction (Fig 5D). When we assessed *IFNB1* gene induction over an infection time-course, up-regulation of transcription occurred as early as 12hpi and remained at or near peak levels through 72hpi (S4A Fig). We also observed induction of *IFNA* transcription, but with delayed kinetics and magnitude as compared to *IFNB1*. *IFNA* transcription was up-regulated at 24hpi during infection with MR-1947, and at 48hpi during infection with the other three strains. These findings are consistent with our recent studies performed in placental macrophages, which showed minimal type I IFN protein secretion, but strong induction at the transcript level during ZIKV infection [34].

Given that RIG-I agonist induced IFNβ secretion, we directly compared *IFNB1* transcript levels in matched moDCs treated with RIG-I agonist or infected with ZIKV PR-2015. RIG-I agonist treatment induced modestly higher, but overall similar levels of *IFNB1* transcription as compared to during ZIKV PR-2015 infection (Fig 5E). Next, to determine if there was impairment in type I IFN protein translation or secretion, we measured type I IFN protein in the supernatant and whole cell lysate from matched samples following RIG-I agonist treatment or infection with ZIKV PR-2015. We hypothesized that if ZIKV blocked protein secretion, but not translation, we would find an accumulation of type I IFN protein in the whole cell lysate. We did not detect IFNβ or IFNα protein in either the supernatants or whole cell lysates above mock levels at either low or high MOI infection (MOI 1 or 10) with ZIKV (Fig 5F). In contrast, both IFNβ and IFNα were observed in the supernatants and whole cell lysates following RIG-I agonist treatment. To determine if ZIKV could actively block type I IFN translation, we treated ZIKV PR-2015-infected moDCs with RIG-I agonist at 48hpi and measured IFNβ protein production. ZIKV infection resulted in an average 2-fold decrease in the induction of IFNβ protein translation as compared to RIG-I agonist alone (Fig 5G). Altogether, our data suggests that ZIKV antagonizes type I IFN translation during infection of human DCs.

Of relevance to our findings, protein kinase R (PKR) is important for maintaining mRNA stability of type I IFN transcripts during infection with certain RNA viruses [52]. In these studies, EMCV infected cells were found to strongly induce *Ifnb1* gene expression, but in the absence of PKR these transcripts lacked poly(A) tails, leading to diminished transcript stability and minimal protein translation. To determine if a similar phenomenon occurs during ZIKV infection of human DCs, we compared *IFNB1* transcript levels after performing cDNA synthesis with random hexamers, which will prime all RNA species, or Olido(dT), which will only prime polyadenylated transcripts. We found no differences in *IFNB1* transcript levels between the two methods, suggesting ZIKV does not influence *IFNB1* transcript stability as a mechanism to inhibit protein translation (S4B Fig).

### ZIKV infection induces an antiviral state within human DCs

**Fig 6:**
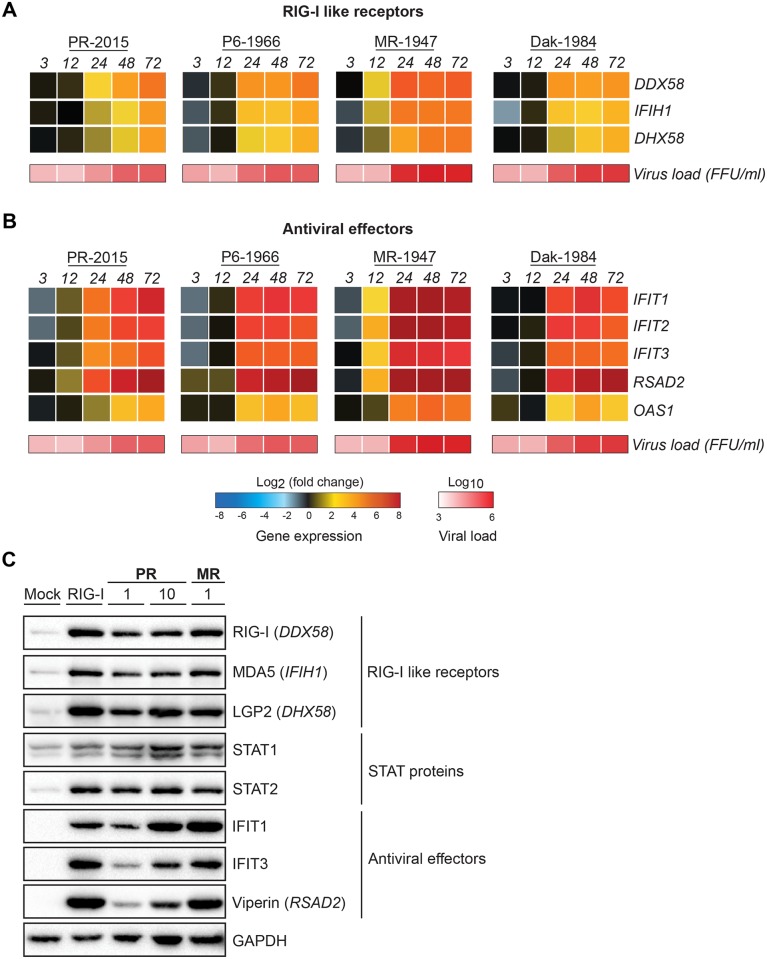
ZIKV infection induces an antiviral state within human DCs. moDCs were infected with ZIKV PR-2015, P6-1966, MR-1947, or Dak-1984 at MOI of 1 (n = 6–8 donors). Cells were collected at indicated hours post-infection and antiviral gene expression was determined by qRT-PCR. Gene expression was normalized to *GAPDH* transcript levels in each respective sample and represented as the averaged log_2_ normalized fold increase above donor and time-point matched uninfected cells. The averaged log_10_ normalized levels of infectious virus (FFU/mL) at each time point is depicted beneath the gene expression heat map. **(A)** RLR gene expression. **(B)** Antiviral effector gene expression. **(C)** moDCs were left untreated (“Mock”), treated with RIG-I agonist (10ng/1e5 cells), or infected with ZIKV PR-2015 (MOIs of 1 and 10) or MR-1947 (MOI 1). After 18hrs of agonist treatment or at 48hpi with ZIKV, whole-cell lysates were collected for western blot analysis of host antiviral effector protein expression. Western blots are shown for a single donor and are representative of data obtained from two donors. See also S5 Fig.

Given the minimal secretion of type I and type III IFNs, we evaluated gene expression of the RLRs and host antiviral effectors. We observed up-regulation of RIG-I (*DDX58*), MDA5 (*IFIH1*), and LGP2 (*DHX58*) in response to PR-2015 and P6-1966 at 24hpi, consistent with increases in virus load (Fig 6A). RLR expression continued to increase through 72hpi. While RLR expression was higher at 24hpi in moDCs infected with P6-1966 as compared to PR-2015, expression peaked at similar levels at 48 and 72hpi, potentially reflecting the slightly enhanced replication kinetics of P6-1966. In contrast to the Asian lineages, MR-1947 exhibited strong RLR up-regulation by 12hpi with peak expression between 24 and 48hpi. Moreover, the magnitude of RLR transcription during peak responses was notably higher for MR-1947 infection. The kinetics of RLR expression during infection with Dak-1984 was more similar to the Asian lineage strains than MR-1947, first increasing at 24hpi. Interestingly, despite reaching a similar overall magnitude of infection as MR-1947, Dak-1984 induced lower RLR transcription at all time-points.

We next evaluated expression of the IFIT gene family members, *OAS1*, and viperin (*RSAD2*), antiviral effectors with known activity against flaviviruses [53]. In moDCs infected with PR-2015, we observed up-regulation of *IFIT1*, *IFIT2* and *IFIT3* beginning at 12hpi, with peak expression between 48 and 72hpi (Fig 6B). P6-1966 infection resulted in slightly delayed IFIT gene induction as compared to PR-2015. Despite this delay, P6-1966 induced stronger IFIT gene expression by 24hpi. We observed similar findings with *RSAD2* expression, with PR-1966 infection inducing lower transcript levels at 12hpi, but increased responses at 24hpi as compared to PR-2015. We found *OAS1* transcripts were up-regulated at 24hpi by both PR-2015 and P6-1966, although to higher levels during P6-1966 infection. MR-1947 infection exhibited enhanced kinetics and magnitude of antiviral effector gene transcription, with *IFIT* family members and *RSAD2* being induced as early as 12hpi. While *OAS1* was up-regulated with similar kinetics to the Asian lineage strains, the magnitude was also notably higher during MR-1947 infection. In general, Dak-1984 was transcriptionally most similar to the Asian lineage viruses, despite higher levels of viral replication during Dak-1984 infection.

Given observed differences in PR-2015 replication between donors, we compared gene expression between donors with “low” or “high” infection (S1B Fig). For all of our RNA samples, we labeled infected cells in parallel for viral E protein, allowing us to stratify our RNA data by the percentage of viral E protein+ cells. Donors with low infection had overall lower expression of RLR, type I IFN, and antiviral effector genes as compared to donors with high infection (S5 Fig). Furthermore, there were no differences in the expression of any of the measured host genes at 3hpi between “low” and “high” infection donors, a time that likely represents basal level expression. Overall, these data show that ZIKV infection is capable of initiating antiviral responses in human DCs, with expression of certain antiviral effector genes being induced rapidly after infection, prior to log phase viral growth.

We next questioned whether the observed up-regulation of antiviral effector genes led to corresponding increases at the protein level, in light of our findings with type I IFN. As expected, overnight stimulation with RIG-I agonist induced up-regulation of the RLRs (RIG-I, MDA5, and LGP2), STAT proteins (STAT1 and STAT2), and multiple proteins directly involved in restriction of viral replication (IFIT1, IFIT3, and viperin) (Fig 6C). Notably, we observed no induction of IFIT1, IFIT3, or viperin in untreated cells. In contrast to impaired translation of type I IFN proteins, infection of moDCs for 48hrs with ZIKV PR-2015 or MR-1947 induced strong up-regulation of the RLRs, STAT proteins, and viral restriction factors to similar levels observed following RIG-I agonist treatment. We observed MOI dependent increases in many cases (STAT1, IFIT1, IFIT3, viperin) following infection with PR-2015 at MOIs of 1 and 10. Similar to what was observed at the transcript level, MR-1947 infection resulted in stronger induction of antiviral proteins as compared to PR-2015 when comparing infections at MOI of 1. This is likely explained by the higher magnitude of infection seen with MR-1947. Together, these findings suggest that ZIKV selectively inhibits in type I IFN protein translation, while translation of other antiviral host proteins remains intact.

### ZIKV replication is blocked by RIG-I, but not type I IFN signaling

**Fig 7:**
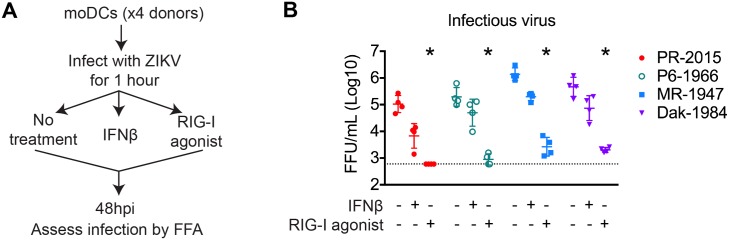
Innate immune signaling restricts ZIKV viral replication within human DCs. **(A)** moDCs were infected with PR-2015, P6-1966, MR-1947, or Dak-1984 at MOI of 1 (n = 4 donors). After viral attachment and entry at 1hpi, cells were treated with RIG-I agonist (10ng/1e5 cells), human IFNβ (100 IU/mL), or left untreated. **(B)** Supernatants were collected at 48hpi and assessed for infectious virus release by FFA. Values for each individual donor are shown with the mean +/- SD. Statistical significance (p< 0.05) was determined using a Friedman test with comparisons made to donor-paired, untreated, ZIKV-infected cells. The assay limit of detection is indicated with a dashed line.

Given our findings that ZIKV infection of moDCs induced an antiviral state, and the importance of RLR and type I IFN signaling in restriction of flavivirus replication [23,26,28,29], we next determined the ability of innate immune signaling pathways to restrict ZIKV replication within human DCs. At 1hpi, we treated infected moDCs with innate immune agonists and assessed viral replication at 48hpi (Fig 7A). To trigger RLR signaling, moDCs were transfected with a highly specific RIG-I agonist, derived from the 3’ UTR of hepatitis C virus [54,55]. To trigger type I IFN signaling, we treated moDCs with 100 IU/mL of recombinant human IFNβ. RIG-I agonist treatment potently blocked ZIKV replication, significantly lowering infectious virus release to levels at or near the assay limit of detection (Fig 7B). Notably, the amount of infectious virus remaining after RIG-I agonist treatment was similar to levels found at 3 and 12hpi (Fig 2A), prior to the log phase viral growth, and may represent residual input virus rather than replicated virus. Importantly, RIG-I agonist treatment restricted replication of all four ZIKV strains. In contrast, type I IFN treatment resulted in only modest, and non-significant decreases in viral replication. Altogether, RLR signaling, but not type I IFN signaling, potently blocks replication of four evolutionarily distinct ZIKV strains.

### ZIKV antagonizes type I IFN signaling by targeting STAT1 and STAT2 phosphorylation

**Fig 8:**
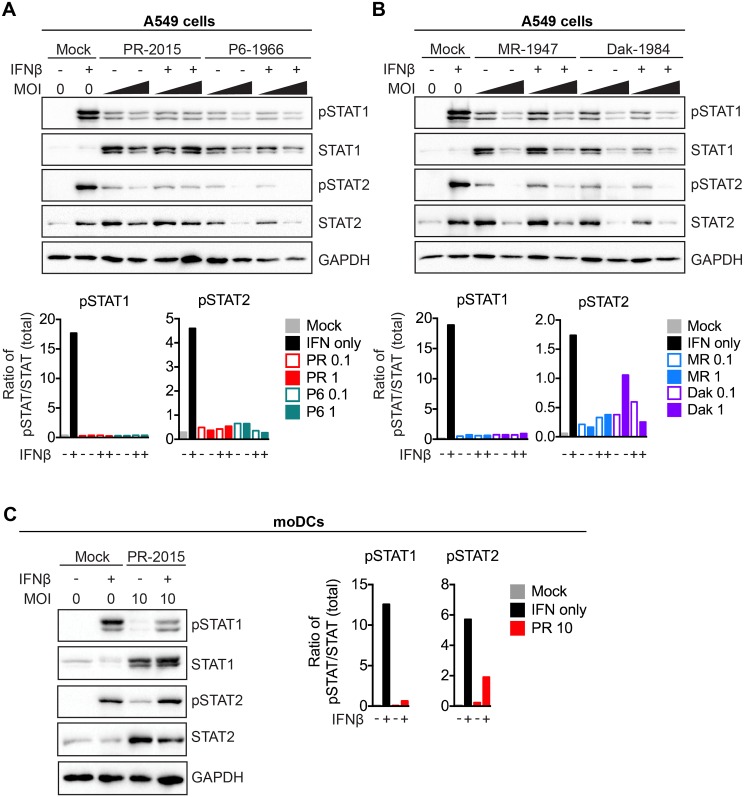
ZIKV antagonizes type I IFN signaling. **(A, B)** A549 cells were infected with PR-2015, P6-1966, MR-1947, or Dak-1984 at MOIs of 0.1 and 1. At 48hpi, cells were pulse treated with 1000 IU/mL of recombinant human IFNβ for 30 minutes and whole-cell lysates were collected for western blot analysis of phospho-STAT1 (Tyr701), phospho-STAT2 (Tyr689), STAT1, STAT2, and GAPDH. Representative blots are shown from one of two independent experiments. Quantitation is shown below the representative blots, where intensity values are represented as the ratio of pSTAT:total STAT protein. **(C)** moDCs were infected with PR-2015 (MOI 10) and STAT1 and STAT2 signaling was assessed as in A and B. Data is representative of three donors from two independent experiments. Quantitation is shown to the right of the representative blots, where intensity values are represented as the ratio of pSTAT:total STAT protein.

Secreted type I IFN binds to the type I IFN receptor, a heterodimeric complex found on the cell surface of almost all nucleated cells, triggering activation of the receptor associated kinases JAK1 and TYK2 [42]. JAK1 and TYK2 phosphorylate and activate the latent transcription factors STAT1 and STAT2, which translocate to the nucleus and associate with IRF-9 to trigger antiviral gene transcription. Most flaviviruses known to infect humans have evolved mechanisms to inhibit type I IFN responses through antagonism of JAK/STAT signaling [56–59]. Given our finding that type I IFN treatment was not effective at blocking ZIKV replication in moDCs, we evaluated the ability of ZIKV to antagonize STAT1 and STAT2. For these studies, we utilized human A549 cells, which have been previously shown to be permissive to ZIKV infection [60] and have been employed to study antiviral innate immune signaling [56,61,62]. We pulse treated uninfected or ZIKV-infected cells (48hpi, MOIs of 0.1 and 1) for 30 minutes with IFNβ (1000 IU/mL) and evaluated phosphorylation of STAT1 (Tyr701) and STAT2 (Tyr689) by western blot. Cells infected with any of the four ZIKV strains did not show enhanced STAT1 or STAT2 phosphorylation above untreated ZIKV-infected cells (Fig 8A and 8B, top panels). Infection alone increased the total levels of STAT1 and STAT2 protein, although notably less so at an MOI of 1 as compared to MOI 0.1. Given the different levels of total STAT proteins between conditions, we calculated the ratio of phosphorylated:total protein to allow for a better comparison of phosphorylation status (Fig 8A and 8B, bottom panels). Indeed, even in instances where ZIKV infection increased total STAT protein levels, the majority remained in an unphosphorylated state. Interestingly, while ZIKV infection alone did induce low levels of STAT1 and STAT2 phosphorylation, in most conditions, there was a notable decrease in phosphorylation at MOIs of 1 as compared to MOIs of 0.1, a finding most profound with the African lineage viruses. We next determined the percentage of ZIKV infected cells at MOIs of 0.1 and 1 using flow cytometry. The percentage of infected cells ranged from 32.7–74% at an MOI of 0.1 and increased to 60.1–87.8% at an MOI of 1 across infection with the four strains (S6 Fig). Of note, we observed higher cytopathic effects and cell death at MOI of 1 as compared to MOI of 0.1 when preparing cells for staining. Given the presence of uninfected cells, even at MOI of 1, it remains possible that the STAT1 and STAT2 phosphorylation observed during infection is from uninfected cells. Nevertheless, this confirms that the majority of cells were ZIKV-infected at the time of pulse treatment with IFNβ and inhibition of type I IFN signaling can be attributed to ZIKV infection.

We next determined whether ZIKV infection antagonizes type I IFN signaling within human DCs. ZIKV PR-2015-infected moDCs (48hpi, MOI 10) were left untreated or pulse treated with IFNβ for 30 minutes and evaluated for STAT1 and STAT2 phosphorylation. Infection with ZIKV PR-2015 in the absence of IFNβ treatment induced minimal STAT1 phosphorylation and low levels of STAT2 phosphorylation, despite notable up-regulation of STAT1 and STAT2 total proteins (Fig 8C, left panel). Treatment of ZIKV infected cells with IFNβ increased phosphorylation of STAT2, and to a lesser extent STAT1, but to notably lower levels than treatment of uninfected cells when accounting for total STAT1 and STAT2 protein levels (Fig 8C, right panel). Combined, this shows that, similar to A549 cells, ZIKV antagonizes the phosphorylation of STAT1 and STAT2 in human DCs.

## Discussion

In our study, we show a contemporary Puerto Rican ZIKV strain, PR-2015, productively infects human DCs with notable donor variation in viral replication, despite no differences in viral binding. Ancestral ZIKV strains of the African (MR-1947 and Dak-1984) and Asian (P6-1966) lineages also infected human DCs. Each strain exhibited unique viral growth curves, with cell death only observed during infection with African lineage strains. We observed minimal up-regulation of co-stimulatory and MHC molecules, inflammatory cytokine secretion, as well as antagonism of type I IFN translation during ZIKV infection, despite notable transcriptional up-regulation of *IFNB1*. Despite this, ZIKV infection induced an antiviral state as noted by strong up-regulation of the RLRs (RIG-I, MDA5, and LGP2), STAT proteins (STAT1 and 2), and antiviral effectors (IFIT1, IFIT3, and viperin). Finally, RIG-I agonist treatment potently restricted ZIKV replication, while type I IFN was significantly less effective due to ZIKV antagonism of STAT1 and STAT2 phosphorylation.

Despite their evolutionary distance [10], minimal attention has been given to studying infection differences between African and contemporary Asian lineage strains. In general, MR-1947 and Dak-1984 replicated with more rapid kinetics and to a higher magnitude than the Asian lineage viruses. The African lineage viruses were also unique in their ability to induce cell death during infection, potentially attributed to their replication characteristics. This raises the possibility that Asian lineage viruses may have adapted to be less cytopathic in DCs, potentially resulting from, or contributing to lower viral replication rates. Alternatively, this phenotype may be partly attributed to the extensive passage history and cell culture adaption of MR-1947, a process known to impact ZIKV and WNV glycosylation patterns [37,63], *in vitro* replication of multiple RNA viruses [63,64], and *in vivo* pathogenesis of hepatitis C virus [65]. In support of this, MR-1947, which has undergone a multitude of passages in suckling mouse brains (S1 Table), replicated with more rapid kinetics than Dak-1984, which has been minimally passaged. Despite differences in kinetics, both viruses reach similar peak infection magnitudes and induced cell death at 72hpi, suggesting cell culture adaption alone does not explain their unique features. Future studies comparing low passaged African and Asian lineage viruses or infectious clone derived viruses [66] are needed to further study differences between these viral genotypes.

While a previous study identified as high as 60% viral E protein-positive cells at 24hr following infection of human moDCs with a French Polynesian strain of ZIKV, we did not observe infection rates this high in our study [67]. This may be explained by differences in ZIKV strains, donor-to-donor variation, or technical differences in virus stock propagation or cell infections. Furthermore, our study did not rely solely on 4G2 staining, which cross-reacts with dengue virus and other flaviviruses, but also included sequence-specific detection of ZIKV RNA to verify infection.

We observed striking variability in viral replication between moDCs generated from different healthy donors. In fact, we found a subset of donors with moDCs that were less susceptible to infection with PR-2015. Although differences in receptor expression or affinity for viral proteins between donors could explain this variability, we found minimal differences in the amount of virus bound to moDCs from different donors. Instead, variability occurred after viral binding, with striking differences in the kinetics and magnitude of viral RNA synthesis, viral E protein staining, and infectious virus release between donors. One plausible explanation for infection differences is that moDCs from less susceptible donors are capable of mounting more rapid and stronger antiviral responses. However, induction of antiviral effector genes was found to be less pronounced in donors with lower viral replication. Moreover, differences in ZIKV replication did not correspond to differential DC activation or pro-inflammatory cytokine release. Of note, susceptibility to PR-2015 replication corresponded to P6-1966, where moDCs with lower PR-2015 infection rates also had lower P6-1966 replication. This raises the possibility that moDCs from some donors are better at controlling ZIKV infection. However, MR-1947 was found to replicate to high levels in moDCs from all donors, even those with low PR-2015 replication. Although this may be related to the aforementioned cell culture adaptation of MR-1947, it is possible that differential host adaption of Asian lineage strains during their evolution has resulted in differences in infection rates. Altogether, complex host factors, such as genetics, metabolism, ER stress, or redox state might explain differential susceptibility to ZIKV infection. Indeed, the collaborative cross, a mouse model of genetic diversity, has recently revealed the importance of host genetics in influencing susceptibility to WNV infection [68]. Similar donor variability in viral replication has also been observed during HIV infection of human monocyte derived macrophages [69]. Although some donor variability in HIV infection was found to correspond with the presence of the CCR5 Δ32 mutation, most of the variability remained unexplained. Influenza A infection of primary human bronchial epithelial cells has also been found to vary notably between donors [70]. Interestingly, cells isolated from obese donors were more susceptible to viral infection, highlighting how complex, non-genetic factors can also influence susceptibility to viral infection at the cellular level. It is interesting to speculate that differential susceptibility of DCs to ZIKV may correspond to pathogenesis during human infection, where 80% of infected individuals are asymptomatic and those with symptoms have differences in clinical presentations.

The minimal activation of DCs following exposure to ZIKV is similar to previous findings with tick-borne encephalitis virus, where DC maturation is inhibited through IRF-1 degradation [24]. Diminished up-regulation of MHC class II and CD40 molecules on splenic CD8α+ DCs was also observed following Japanese encephalitis virus infection in mice [25]. In contrast to our findings with ZIKV, infection of human moDCs with the yellow fever virus vaccine strain, YF-17D, promotes DC maturation [71]. The ability of YF-17D to activate human DCs may be explained through the loss of a viral antagonist during its attenuation process, or could represent a unique behavior of certain flaviviruses. Indeed, infection of human moDCs with a pathogenic dengue virus serotype 2 strain also promotes the up-regulation of co-stimulatory and MHC molecules, along with pro-inflammatory cytokine secretion [72]. Combined, this work suggests members of *Flaviviridae* have evolved complementary, as well as unique strategies of targeting DCs to subvert the pressures of host immunity.

While infection with all four ZIKV strains induced type I IFN mRNA transcription, we detected minimal translation of type I or III IFN proteins. This was in contrast to RIG-I agonist treatment, which induced translation of both type I and III IFN proteins, despite similar levels of *IFNB1* transcription as observed during ZIKV infection. Indeed, ZIKV infection diminished RIG-I agonist-induced type I IFN production, suggesting ZIKV directly antagonizes type I IFN translation. We also observed a minor 2–3 fold enhancement in viral infection when type I IFN signaling was inhibited by antibody-mediated receptor blockade, further indicating type I IFN is secreted at minimal levels during ZIKV infection. Previous work with dengue virus observed secretion of IFNα protein during infection of human moDCs, suggesting our findings might be unique to ZIKV infection [72]. Despite the antagonism of type I or III IFN production, ZIKV infection up-regulated the expression of the RLRs, STAT proteins, and multiple antiviral effector proteins to similar levels observed following RIG-I agonist treatment. This suggests that the block in type I IFN translation is selective, and that much of the antiviral response induced during ZIKV infection of human DCs occurs independent of type I IFN signaling. Indeed, in the context of WNV infection, multiple antiviral effector genes are induced through an IFN-independent, RLR signaling-dependent manner [26].

Recent work from multiple groups has analyzed ZIKV infection and immune responses from human clinical samples and in a variety of human cell types. During the acute phase of human ZIKV infection, multiple pro-inflammatory cytokines are increased within the blood, although the cellular sources of these responses remain unknown [73]. Our findings suggest that infected DCs may not be an important source of these pro-inflammatory cytokines. In contrast to our work with DCs, ZIKV infection of A549 cells has been shown to induce IFNβ secretion, further suggesting cells other than DCs may be responsible for inducing inflammatory responses during ZIKV infection [60]. Indeed, *ex vivo* infection of primary human skin fibroblasts was found to induce transcriptional up-regulation of multiple pro-inflammatory mediators, although protein secretion was not explored [67]. In regards to congenital ZIKV infection, recent work has found both human fetal neural progenitor cells and placental Hofbauer cells are poorly immunogenic, similar to our findings with adult DCs [34,74]. In contrast, human embryonic cranial neural crest cells secrete cytokines following ZIKV infection at levels that were found to be harmful for neurodevelopment [75]. Together, different target cells of ZIKV have varying capacities to induce pro-inflammatory cytokine responses and further study is needed to determine the cell types responsible for initiating inflammatory responses during human infection.

Recent work has revealed that the NS5 protein of both MR-1947 and PR-2015 promotes the degradation of human STAT2 protein during infection, allowing ZIKV to evade type I IFN signaling downstream of the type I IFN receptor [31]. In agreement with this work, we found that while RIG-I agonist treatment potently restricted viral replication, type I IFN treatment was significantly less effective at blocking ZIKV infection. Mechanistically, we found infection with both contemporary and ancestral strains of ZIKV blocked phosphorylation of STAT1 and STAT2 downstream of type I IFN signaling in both human DCs and A549 cells. In contrast to previous findings, we did not observe significant STAT2 degradation in either human DCs or A549 cells [31]. In fact, in most cases, we observed up-regulation of STAT2 protein during ZIKV infection. One possibility for this discrepancy may be differences in the cell types used between the studies. Grant et al performed studies in Vero and HEK 293 cells, while we conducted experiments in A549 cells and primary human DCs. We also used lower MOIs (0.1 and 1) for our A549 cell line infections than in their studies (MOI 5, 10, and 20) and did not perform viral protein overexpression studies. Although we did use an MOI of 10 for some of our DC work, the magnitude of infection is significant lower than in Vero or HEK 293 cells and such differences in cell infectivity could also explain our differing findings. Nevertheless, we find that ZIKV antagonizes the type I IFN signaling pathway through blockade of STAT1 and STAT2 phosphorylation.

The ability of RIG-I agonist to efficiently block ZIKV replication is most likely attributed to an IFN-independent induction of antiviral effector molecules [26,43]. Our observations that ZIKV infection induces an antiviral state in moDCs, despite viral antagonism of type I IFN responses, further suggests IFN-independent signaling pathways, such as RLR signaling through MAVS, are important for restriction of ZIKV replication. The ability of RIG-I agonist to potently restrict ZIKV replication across all four strains highlights the RLR signaling pathway as a potential target for antiviral therapy. Of note, small molecule agonists of the RLR pathway have gained recent attention as potential candidate vaccine adjuvants [76,77] and for use in broad-spectrum antiviral therapy, including proof-of-principle studies showing potent activity against multiple flaviviruses [43,78].

In summary, our work shows that human DCs are productively infected by currently circulating (PR-2015) and ancestral (P6-1966, MR-1947, and Dak-1984) strains of ZIKV. Each ZIKV strain exhibited unique replication kinetics and downstream effects on human DCs, including a unique ability of African lineage viruses to induce cell death. There was notable donor variability in viral replication across the ZIKV strains, highlighting the importance of both host and viral factors in influencing susceptibility during infection. We observed minimal DC activation or secretion of inflammatory cytokines, as well as viral antagonism of type I IFN translation, despite strong induction of *IFNB1* at the RNA transcript level. Nevertheless, ZIKV-infected moDCs induced an antiviral state as noted by strong up-regulation of multiple antiviral effectors. RIG-I agonist treatment potently restricted ZIKV replication in human DCs, while type I IFN treatment had minimal effects. Mechanistically, all strains of ZIKV antagonized type I IFN-mediated phosphorylation of STAT1 and STAT2. Combined, our findings show that ZIKV efficiently evades type I IFN responses, but RLR signaling remains functional and may be a target for antiviral therapy in humans.

## Materials and Methods

### Ethics statement

Human peripheral blood mononuclear cells (PBMCs) were obtained from healthy donors in accordance with the Emory University Institutional review board according to IRB protocol IRB00045821.

### Virus stocks

Zika virus strains PRVABC59 (PR-2015), P6-740 (P6-1966), MR-766 (MR-1947), and DakAr 41524 (Dak-1984) were obtained from the Centers for Disease Control and Prevention. All strains were passaged once in Vero cells cultured in MEM (Life Technologies Gibco) supplemented with 10% FBS (Optima, Atlanta Biologics) to generate working viral stocks. Viral stocks were titrated by plaque assay on Vero cells as previously described [34] and stored at -80°C in MEM with 20% FBS.

### Viral stock sequencing and genome annotation

The Zika virus isolates in this study were subjected to whole genome sequencing using previously described methods [79]. Briefly, total viral RNA was subjected to next generation sequencing library construction with random hexamer-based priming methods. Libraries were sequenced on the Illumina MiSeq platform and genome assembly was performed with CLC Bio (clc_ref_assemble_long v. 3.22.55705). Viral genome annotation was performed with VIGOR [80]. The Genbank accession numbers are: KX601166.1 (Zika virus strain ZIKV/Aedes africanus/SEN/DakAr41524/1984); KX601167.1 (Zika virus strain ZIKV/Aedes sp./MYS/P6-740/1966); KX601168.1 (Zika virus strain ZIKV/Homo Sapiens/PRI/PRVABC59/2015); KX601169.1 (Zika virus strain ZIKV/Macaca mulatta/UGA/MR-766/1947).

### Cells

Vero and A549 cells were obtained from ATCC and maintained in complete DMEM (DMEM medium [Corning] supplemented with 10% fetal bovine serum [Optima, Atlanta Biologics], 2mM L-Glutamine [Corning], 1mM HEPES [Corning], 1mM sodium pyruvate [Corning], 1x MEM Non-essential Amino Acids [Corning], and 1x Antibiotics/Antimycotics [Corning]). moDCs, monocytes, mDCs and pDCs were maintained in complete RPMI (RPMI 1640 medium [Corning] supplemented with 10% fetal bovine serum [Optima, Atlanta Biologics], 2mM L-Glutamine [Corning], 1mM Sodium Pyruvate [Corning], 1x MEM Non-essential Amino Acids [Corning], and 1x Antibiotics/Antimycotics [Corning]).

### Primary cell isolation

PBMCs were isolated from freshly obtained healthy donor peripheral blood using lymphocyte separation media (MP Biomedicals or StemCell Technologies) per manufacturer’s instructions. CD14+ monocytes were magnetically purified by positive selection using the EasySep Human CD14 Positive Selection Kit (Stem Cell Technologies) per manufacturer’s instructions. CD14+ monocytes were resuspended in complete RPMI medium with 100ng/mL each of recombinant human IL-4 and GM-CSF (PeproTech) at a cell density of 2e6 cells/mL. Spent media and non-adherent cells were removed 24 hours later and replaced with fresh media and cytokines. Suspension cells were harvested 5–6 days later for use in experiments. moDCs were consistently CD14-, CD11c+, HLA-DR+, DC-SIGN+, and CD1a+ by flow cytometry. To obtain mDCs and pDCs, monocytes were removed by positive selection using CD14 microbeads (Miltenyi Biotech) and the CD14- fraction was enriched for DCs using a human Pan-DC Enrichment Kit (Miltenyi Biotech). Enriched cells were surface stained to identify mDCs and pDCs for fluorescence activated cell sorting (FACS). Within the lineage-negative HLA-DR+ population, CD1c+ mDC1 and CD141+ mDC2 were collected together as CD11c+ mDCs, and CD123+ cells were collected as pDCs. Purity of microbead-sorted monocytes and FACS-sorted DC populations was >95%. Monocytes, mDCs and pDCs were maintained in complete RPMI medium. mDCs were cultured in the presence of human GM-CSF (2 ng/ml). pDC were cultured in the presence of human IL-3 (10 ng/ml).

### Cell culture infections

moDCs were harvested after 5–6 days of differentiation and resuspended in complete RPMI (without GM-CSF or IL-4) at 1e5 cells per well of a 96-well V-bottom plate for infections. moDCs, monocytes, mDCs, and pDCs were infected with the indicated ZIKV strain at MOIs of 1 or 10 (based on Vero cell titer) for 1hr at 37°C. After 1hr, virus inoculum was washed off and cells were resuspended in 200μL fresh media and incubated at 37°C for 3-72hr.

### Viral binding assay

moDCs were infected with ZIKV at MOI of 1 for 1hr on ice and washed 4x with cold PBS (S1C Fig). To remove bound virus, cells were then incubated with trypsin for 60 minutes on ice and washed 4x with cold PBS. Bound virus was quantitated by qRT-PCR for ZIKV RNA.

### Agonist stimulation of moDCs

After 5–6 days of differentiation, moDCs were harvested and plated at 1e5 cells per well of a 96-well V-bottom plate in complete RPMI medium (without GM-CSF or IL-4) and stimulated with innate immune agonists. To stimulate RIG-I signaling, 10ng of a highly specific RIG-I agonist derived from the 3’-UTR of hepatitis C virus [55] was transfected per 1e5 cells using an mRNA transfection kit (Mirus). To stimulate type I IFN signaling, 1e5 cells were cultured in 200μL complete RPMI media in the presence of 100 IU/mL of human recombinant IFNβ (PBL Assay Science). To inhibit endogenous type I IFN signaling, 1e5 cells were cultured in 200μL complete RPMI media in the presence of 1.25μg/mL anti-human Interferon-α/β Receptor Chain 2 (clone MMHAR-2, EMD Milipore) blocking monoclonal antibody.

### Focus forming assay (FFA)

Supernatants collected from moDCs were diluted in DMEM supplemented with 1% FBS and used to infect Vero cells for 1hr at 37°C. Cells and inoculum were overlaid with methylcellulose (OptiMEM [Corning], 1% Antibiotic/Antimycotic [Corning], 2% FBS, and 2% methylcellulose [Sigma Aldrich]) and incubated for 72hr at 37°C. Cells were washed with PBS to remove methylcellulose and fixed with a 1:1 methanol:acetone mixture for 30min. Cells were blocked with 5% milk in PBS at RT for 20min. Cells were incubated with primary antibody (mouse 4G2 monoclonal antibody) at 1μg/mL in 5% milk in PBS for 2hr at RT. Cells were incubated with secondary antibody (HRP-conjugated goat anti-mouse IgG) diluted 1:3000 in 5% milk in PBS for 1hr at RT. Foci were developed with TrueBlue Peroxidase Substrate (KPL). Plates were read on a CTL-ImmunoSpot S6 Micro Analyzer.

### Quantitative reverse transcription-PCR (qRT-PCR)

Total RNA was purified from 1e5 moDCs using the Quick-RNA MiniPrep kit (Zymo Research) per the manufacturer’s instructions. Purified RNA was reverse transcribed using the High Capacity cDNA Reverse Transcription Kit (Applied Biosystems) using random hexamers. For quantitation of viral RNA and host gene expression, qRT-PCR was performed as previously described [34].

### Sequence alignment

All pairwise alignments between ZIKV PR-2015, P6-1966, MR-1947, and Dak-1984 were performed using MegAlign and the Jotun Hein method. For calculations of nucleotide sequence similarity indices, the Martinez/Needleman-Wunsch method was used, and the parameters included a minimum match of 9, gap penalty of 1.1, and gap length penalty of 0.33.

### Flow cytometry and ImageStream analysis

The following mouse anti-human antibodies were purchased from BioLegend or Becton Dickinson: CD11c (B-Ly6), HLA-DR (G46-6), CD1a (HI149), CD209 (9E9A8), CD14 (M5E2), CD80 (2D10), CD86 (IT2.2), and CD40 (5C3). Unconjugated monoclonal 4G2 antibody was kindly provided by Dr. Jens Wrammert (Emory University) and conjugated to APC (Novus Lightning-Link). Following 10min of Fc receptor blockade on ice (Human TruStain FcX, BioLegend), 1e5 cells were sequentially stained for surface markers and viability (Ghost Dye Red 780, Tonbo Biosciences) for 20min on ice. For intracellular staining of ZIKV E protein, cells were fixed and permeabilized (Foxp3/Transcription Factor Staining Buffer Kit, Tonbo Biosciences), blocked for 10 minutes (Human TruStain FcX and 10% normal mouse serum), and stained with 4G2-APC for 20min at room temperature. Multi-color flow cytometry acquisition was performed on a BD LSR II and data was analyzed using FlowJo version 10. ImageStream data acquisition was performed on an ImageStream X Mark II and data was analyzed using Amnis IDEAS software. Monocytes, mDCs and pDCs were stained for viability using Zombie Aqua Fixable Viability Kit in protein-free buffer. Cells for surface staining were suspended in 10% FCS/PBS and incubated with antibodies for 20min at 4°C. Cells were washed, fixed with BD Fix buffer, and acquired on a BD LSR II with all analysis performed using FlowJo version 10.

### Multiplex bead array

Cytokine analysis was performed on supernatants obtained from 1e5 moDCs following the indicated treatment conditions using a human magnetic 25-plex panel (ThermoScientific) and a custom magnetic 3-plex panel with human IFNβ, IFNα, and IFNλ1 (eBioscience) per the manufacturer’s instructions, and read on a Luminex 100 Analyzer. For cytokine analysis within whole cell lysates, 1e5 moDCs were collected in modified radioimmunoprecipitation assay buffer (10 mM Tris [pH 7.5], 150 mM NaCl, 1% sodium deoxycholate, and 1% Triton X-100) supplemented with Halt Protease Inhibitor Cocktail (ThermoFisher) and diluted 1:5 prior to luminex analysis. Culture supernatants from monocytes, mDCs or pDCs were analyzed for cytokine and chemokines using Cytokine Bead Array (CBA) kits (BD Biosciences, San Diego, US) per the manufacturer’s instructions. Cytokines analyzed included: GM-CSF, TNF-α, IL-4, IL-6, MIP-1α, IL-8, IL-15, IL-2R, IP-10, MIP-1β, Eotaxin, RANTES, MIG, IL-1RA, IL-12 (p40/p70) IL-13, IFN-γ, MCP-1, IL-7, IL-17, IL-10, IL-5, IL-2, IL-1β, IFNα, IFNβ, and IFNλ1.

### Western blot analysis

STAT1 and STAT2 signaling was studied in A549 cells as previously described [61]. Briefly, A549 cells were infected with the indicated ZIKV strain at an MOI of 0.1 and 1 (based on Vero cell titration). At 48hpi, cells were pulse treated with 1000 IU/mL of recombinant human IFNβ (PBL Assay Science) for 30 minutes and whole-cell lysates were collected in modified radioimmunoprecipitation assay buffer supplemented with Halt Protease Inhibitor Cocktail (ThermoFisher) and phosphatase inhibitor cocktail II (Calbiochem). Western blot analysis was performed to detect STAT1 phosphotyrosine residue 701 (Cell Signaling), total STAT1 (Cell Signaling), STAT2 phosphotyrosine residue 689 (Upstate, EMD Milipore), total STAT2 (Cell Signaling), and glyceraldehyde 3-phosphate dehydrogenase (GAPDH; Cell Signaling). Protein expression levels were quantified using Image Lab software. For analysis of antiviral effector proteins within human moDCs, 4e5 cells were used per condition and protein lysates were collected as described for A549 cells. The following antibodies were obtained from Cell Signaling: RIG-I, MDA5, LGP2, STAT1, STAT2, IFIT1, viperin, and GAPDH. The IFIT3 antibody was kindly provided by Dr. G. Sen.

## Supporting Information

S1 Fig Related to Fig 1, ZIKV PR-2015 productively infects moDCs. **(A)** moDCs were mock-infected, infected with ZIKV PR-2015, or UV-inactivated PR-2015 (“UV ZIKV”) at MOI of 1 and the percentage of infected cells was assessed by ZIKV E protein staining. ZIKV PR-2015 was inactivated by exposure to ultraviolet (UV) light for 1hr. hpi, hours post-infection. **(B)** Donors were stratified into “high” and “low” infection. **(C)** Experimental outline for ZIKV binding assay.
(TIF)

S2 Fig Related to Figs 2–7, ZIKV strains used in this study. **(A)** Experimental outline used to obtain data in Fig 2. moDCs were generated from healthy donors and infected with all four strains of ZIKV (n = 6 donors). We performed parallel analysis of viral RNA, infectious virus release, and viral E protein staining from each of these samples. **(B)** Viral RNA was detected by qRT-PCR for ZIKV E protein RNA. Gene expression is shown as relative expression after normalization to *GAPDH* levels in each respective sample (n = 6 donors). **(C)** Representative FFA staining for the different ZIKV stains. Serial dilutions are indicated across the top.
(TIF)

S3 Fig Related to Fig 3, ZIKV PR-2015 does not induce activation of human blood monocytes or DC subsets. **(A)** moDCs were left untreated (“Mock”) or treated with RIG-I agonist (10ng/1e5 cells) for 24hrs. Cells were labeled for indicated DC activation markers and surface expression was quantitated by flow cytometry. Values are represented as the average median fluorescence intensity (MFI) of three technical replicates. Error bars represent the SD. Statistical significance was determined as P<0.05 by a Mann Whitney U test. **(B)** Monocytes, **(C)** myeloid DCs (mDCs) and **(D)** plasmacytoid DCs (pDCs) were left untreated (“Mock”) or infected with PR-2015 at MOI of 1 (n = 5 donors). Cells were collected at 24hpi and labeled for indicated DC activation markers. Surface expression was quantitated by flow cytometry. Values for each donor are represented as the median fluorescence intensity (MFI), with mock and ZIKV infected samples from the same donor connected with a line. Statistical significance was determined as p<0.05 using a Wilcoxon signed-rank test **(B-D)**. Of note, no values were statistically significant in panels B-D.
(TIF)

S4 Fig Related to Fig 5, ZIKV induces type I IFN gene transcription. **(A)** moDCs were infected with ZIKV PR-2015, P6-1966, MR-1947, or Dak-1984 at MOI of 1 (n = 6–8 donors). Cells were collected at indicated hours post-infection and antiviral gene expression was determined by qRT-PCR. **(B)** moDCs were treated with RIG-I agonist (10ng/1e5 cells) or virally infected with ZIKV PR-2015 at MOI of 1 (n = 4 donors). At 48hpi, RNA was isolated, reverse transcribed using either random hexamer or Oligo(dT) primers, and *IFNB1* expression was determined by qRT-PCR. All gene expression was normalized to *GAPDH* transcript levels in each respective sample and represented as the log_2_ normalized fold increase above donor- and time point-matched uninfected cells. Error bars represent the mean +/- SD.
(TIF)

S5 Fig Related to Figs 5 and 6, Antiviral effector gene expression corresponds with viral replication. moDCs from eight donors infected with ZIKV PR-2015 were separated into “high infection” (5 donors) and “low infection” (3 donors) on the basis of E protein staining as assessed by flow cytometry (see Fig 1C). Antiviral gene expression was determined by qRT-PCR. Gene expression was normalized to *GAPDH* transcript levels in each respective sample and represented as the averaged log_2_ normalized fold increase above donor- and time point-matched uninfected cells. Error bars represent the mean +/- SD.
(TIF)

S6 Fig Related to Fig 8, ZIKV antagonizes type I IFN signaling. Representative flow plots of A549 cells infected with indicated ZIKV strain at MOI of 0.1 or 1 for 48hrs and labeled for the presence of viral E protein. Data is representative of two independent experiments.
(TIF)

S1 Table Related to Figs 1 and 2, ZIKV isolates used in this study. Information about the ZIKV strains used throughout these studies, nucleotide similarity between coding regions of ZIKV strain genomes, and amino acid differences between viral proteins of ZIKV strains. CDS- coding DNA sequence, V- Vero cell, SM- suckling mouse brain, Ap61- Aedes pseudoscutellaris cell line, C6- Aedes albopictus clone C6/36 cell line.
(PDF)

S2 Table Related to Fig 4, Cytokine production by monocyte derived DCs (moDCs). moDCs were left untreated (“Mock”), transfected with RIG-I agonist (10ng/1e5 cells), or infected with ZIKV PR-2015, P6-1966, MR-1947, or Dak-1984 at MOI of 1 (n = 7 donors). Cytokine levels in the supernatants were determined by multiplex bead array at 24hrs post-agonist transfection or 48hrs post-infection. All values are represented in “pg/mL”. Cytokine levels that were below the lower limit of detection are indicated as not detected or “ND”. LLOQ, lower limit of quantitation.
(PDF)

S3 Table Related to Fig 4, Cytokine production by human blood monocytes. Monocytes were left untreated (“Mock”), treated with LPS (100ng/mL), or infected with ZIKV PR-2015 at MOI of 1 (n = 4–5 donors). Cytokine levels in the supernatants were determined by multiplex bead array 24hrs later. Cytokines that were not assayed are indicated as “-“.
(PDF)

S4 Table Related to Fig 4, Cytokine production by human blood myeloid DCs (mDCs). mDCs were left untreated (“Mock”), treated with LPS (100ng/mL), or infected with ZIKV PR-2015 at MOI of 1 (n = 4–5 donors). Cytokine levels in the supernatants were determined by multiplex bead array 24hrs later. Cytokines that were not assayed are indicated as “-“.
(PDF)

S5 Table Related to Fig 4. Cytokine production by human blood plasmacytoid DCs (pDCs). pDCs were left untreated (“Mock”), treated with R848 (1μg/mL) or infected with ZIKV PR-2015 at MOI of 1 (n = 4–5 donors). Cytokine levels in the supernatants were determined by multiplex bead array 24hrs later.
(PDF)
