# Supplementary figures and images for: Zika Virus Antagonizes Type I Interferon Responses during Infection of Human Dendritic Cells
Source: PLoS Pathog. 2017 Feb 2;13(2):e1006164. doi: 10.1371/journal.ppat.1006164 (PMC5289613; doi:10.1371/journal.ppat.1006164)

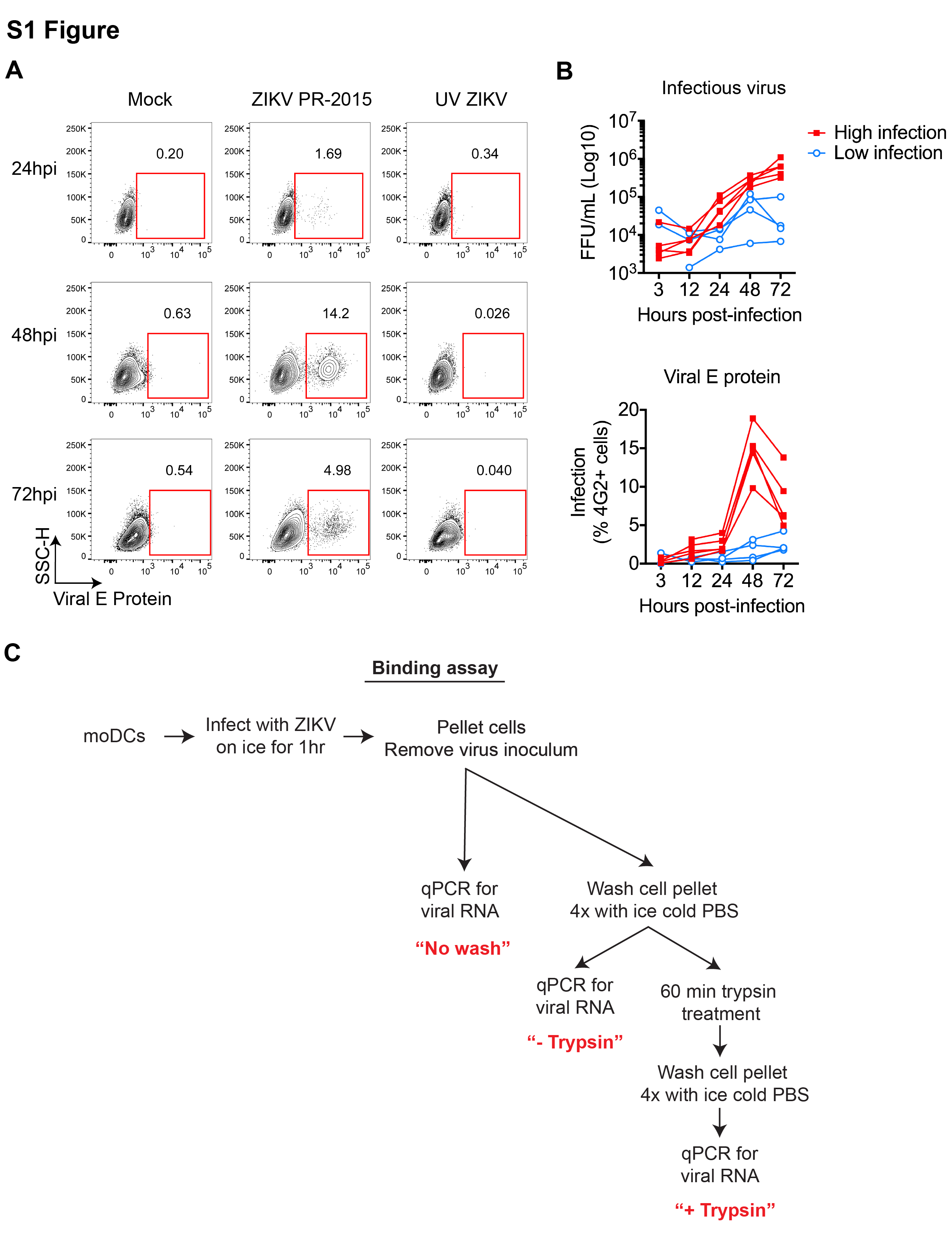

Supplement: S1 Fig — (A) moDCs were mock-infected, infected with ZIKV PR-2015, or UV-inactivated PR-2015 (“UV ZIKV”) at MOI of 1 and the percentage of infected cells was assessed by ZIKV E protein staining. ZIKV PR-2015 was inactivated by exposure to ultraviolet (UV) light for 1hr. hpi, hours post-infection. (B) Donors were stratified into “high” and “low” infection. (C) Experimental outline for ZIKV binding assay. (TIF) [file ppat.1006164.s001.tif]

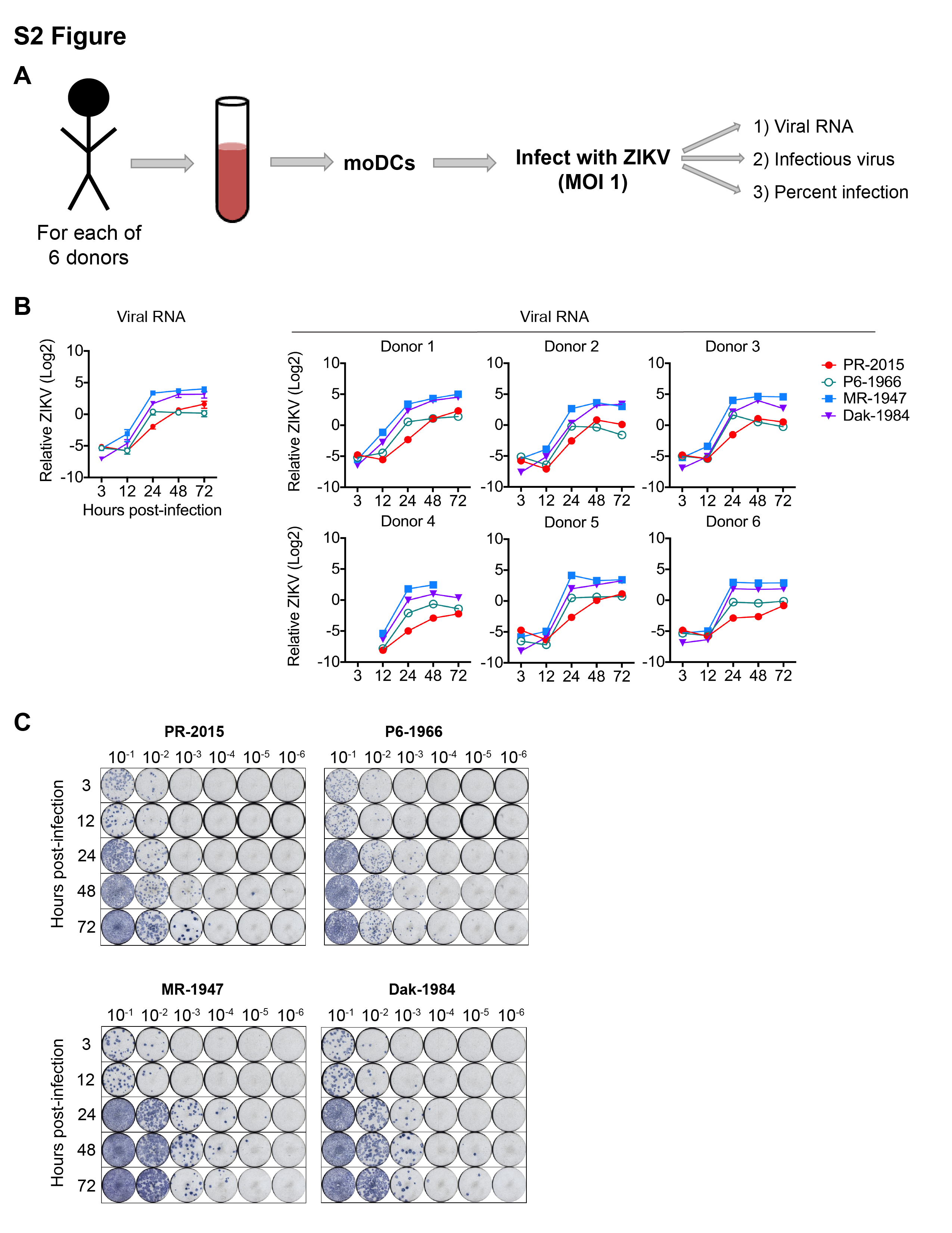

Supplement: S2 Fig — (A) Experimental outline used to obtain data in Fig 2. moDCs were generated from healthy donors and infected with all four strains of ZIKV (n = 6 donors). We performed parallel analysis of viral RNA, infectious virus release, and viral E protein staining from each of these samples. (B) Viral RNA was detected by qRT-PCR for ZIKV E protein RNA. Gene expression is shown as relative expression after normalization to GAPDH levels in each respective sample (n = 6 donors). (C) Representative FFA staining for the different ZIKV stains. Serial dilutions are indicated across the top. (TIF) [file ppat.1006164.s002.tif]

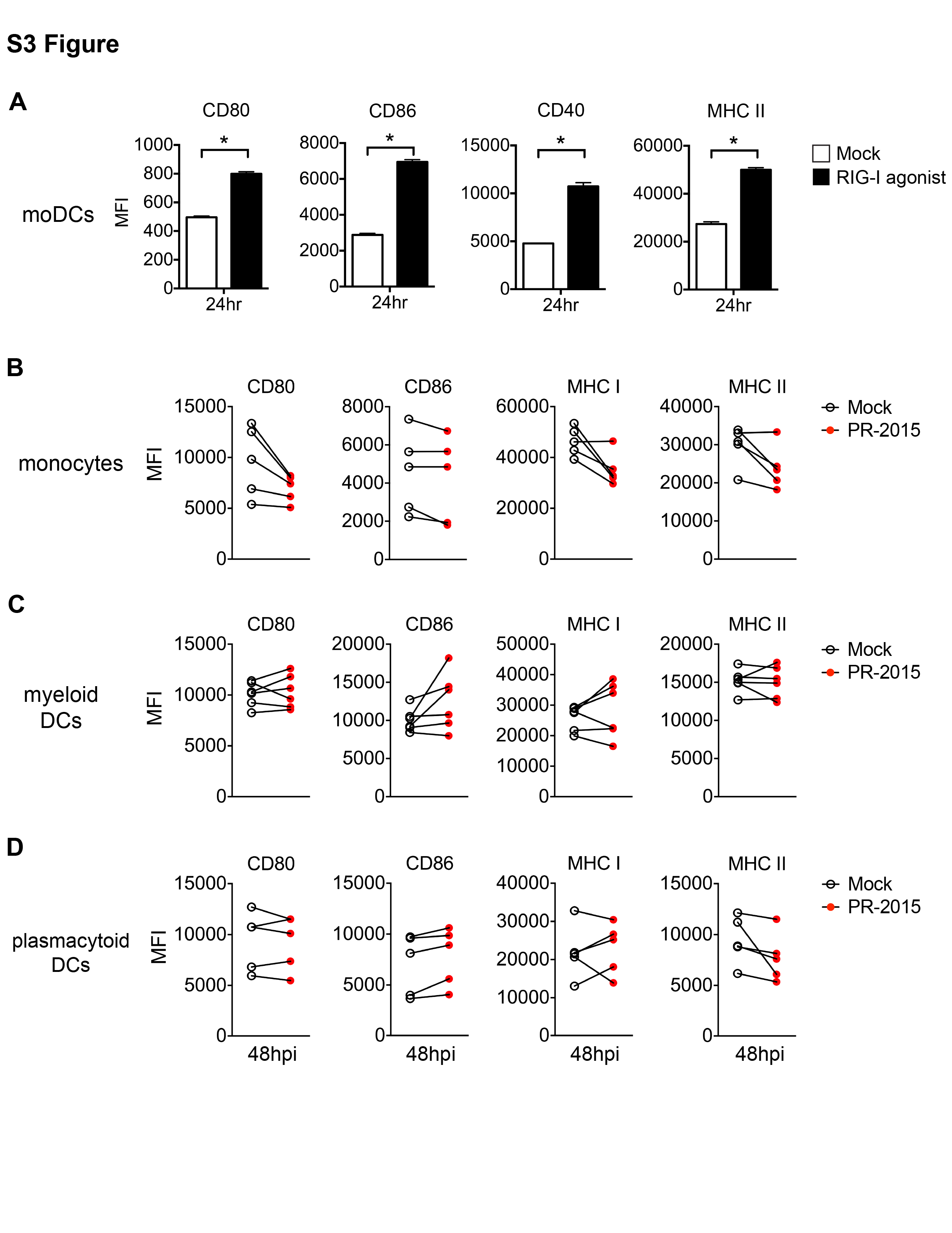

Supplement: S3 Fig — (A) moDCs were left untreated (“Mock”) or treated with RIG-I agonist (10ng/1e5 cells) for 24hrs. Cells were labeled for indicated DC activation markers and surface expression was quantitated by flow cytometry. Values are represented as the average median fluorescence intensity (MFI) of three technical replicates. Error bars represent the SD. Statistical significance was determined as P<0.05 by a Mann Whitney U test. (B) Monocytes, (C) myeloid DCs (mDCs) and (D) plasmacytoid DCs (pDCs) were left untreated (“Mock”) or infected with PR-2015 at MOI of 1 (n = 5 donors). Cells were collected at 24hpi and labeled for indicated DC activation markers. Surface expression was quantitated by flow cytometry. Values for each donor are represented as the median fluorescence intensity (MFI), with mock and ZIKV infected samples from the same donor connected with a line. Statistical significance was determined as p<0.05 using a Wilcoxon signed-rank test (B-D). Of note, no values were statistically significant in panels B-D. (TIF) [file ppat.1006164.s003.tif]

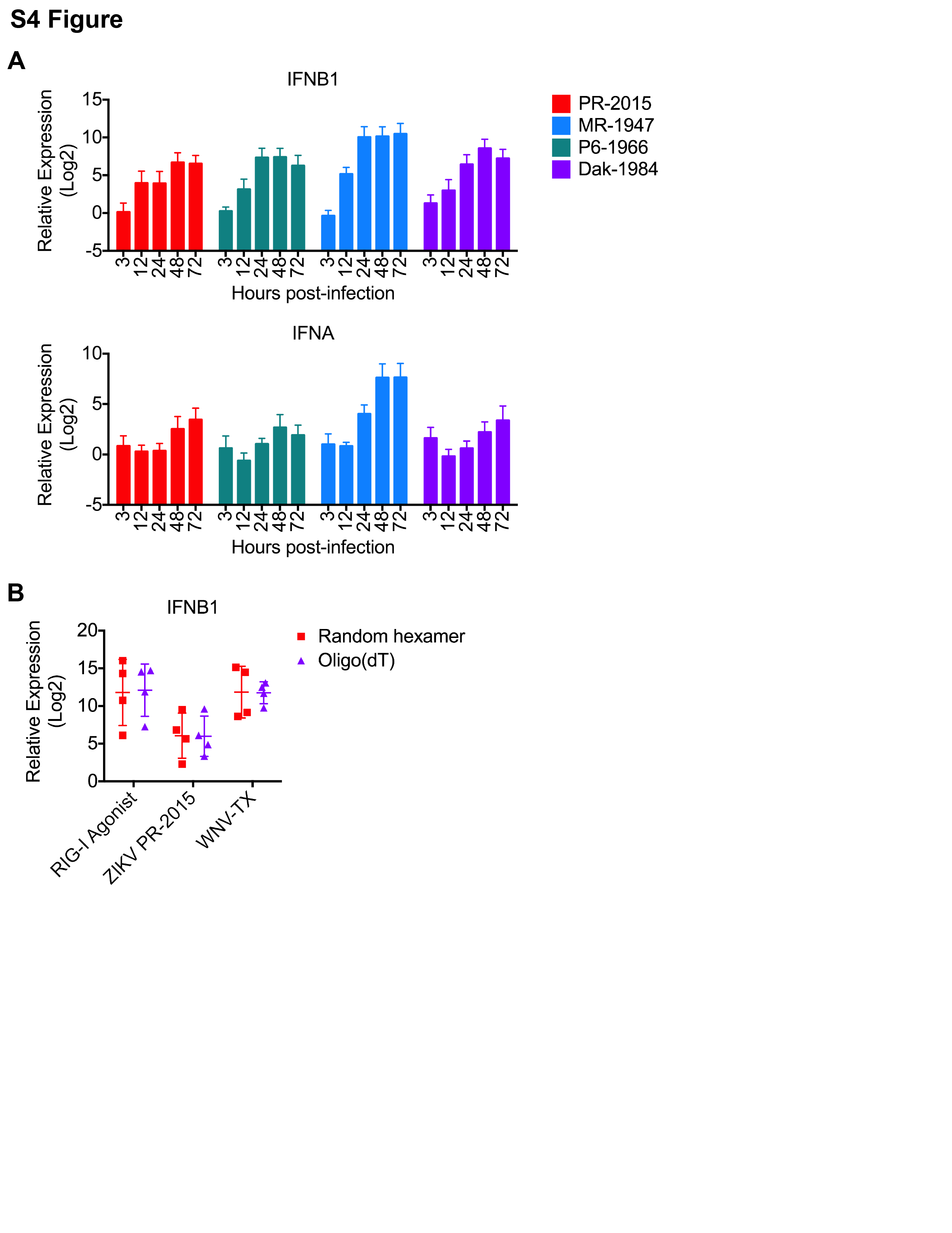

Supplement: S4 Fig — (A) moDCs were infected with ZIKV PR-2015, P6-1966, MR-1947, or Dak-1984 at MOI of 1 (n = 6–8 donors). Cells were collected at indicated hours post-infection and antiviral gene expression was determined by qRT-PCR. (B) moDCs were treated with RIG-I agonist (10ng/1e5 cells) or virally infected with ZIKV PR-2015 at MOI of 1 (n = 4 donors). At 48hpi, RNA was isolated, reverse transcribed using either random hexamer or Oligo(dT) primers, and IFNB1 expression was determined by qRT-PCR. All gene expression was normalized to GAPDH transcript levels in each respective sample and represented as the log2 normalized fold increase above donor- and time point-matched uninfected cells. Error bars represent the mean +/- SD. (TIF) [file ppat.1006164.s004.tif]

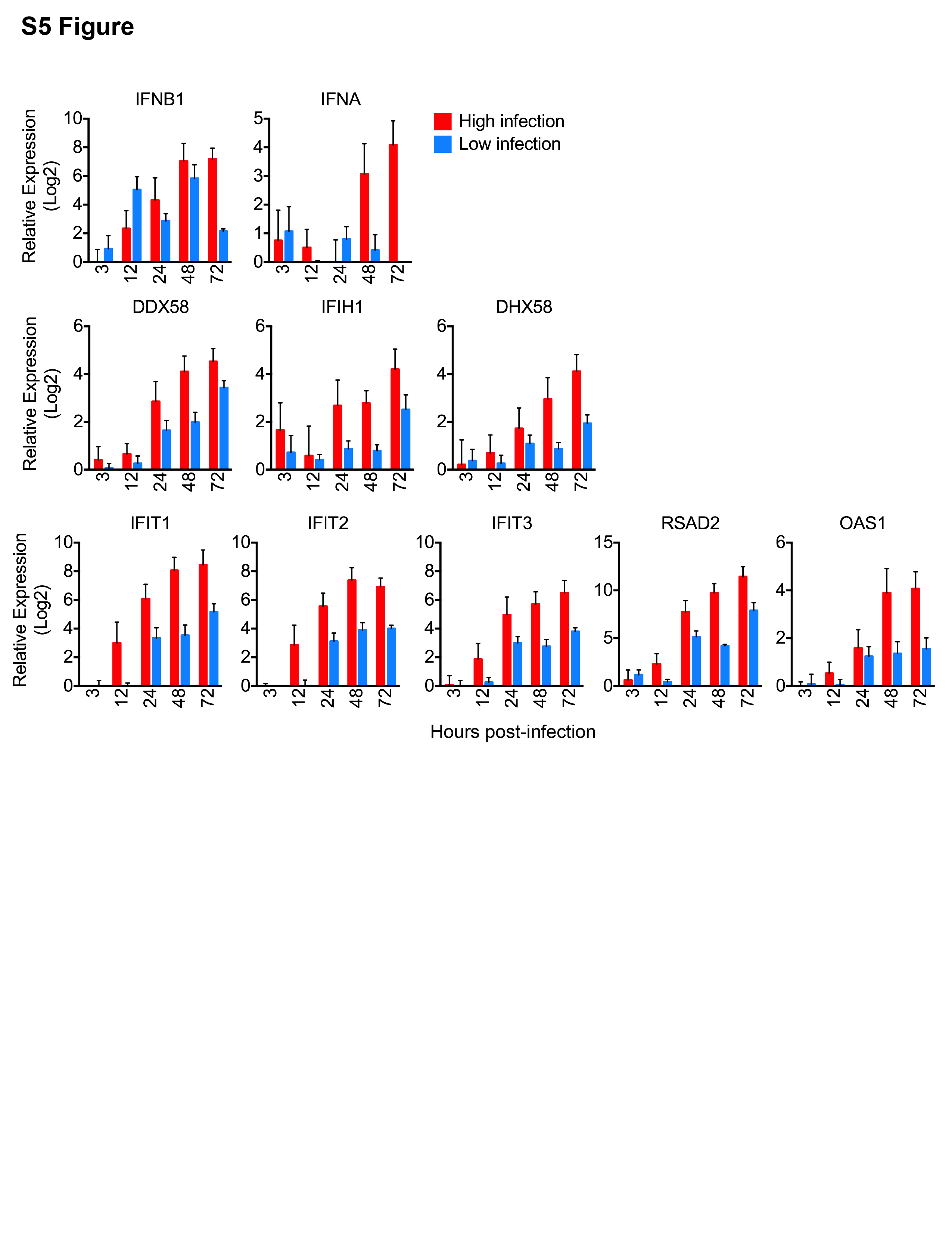

Supplement: S5 Fig — moDCs from eight donors infected with ZIKV PR-2015 were separated into “high infection” (5 donors) and “low infection” (3 donors) on the basis of E protein staining as assessed by flow cytometry (see Fig 1C). Antiviral gene expression was determined by qRT-PCR. Gene expression was normalized to GAPDH transcript levels in each respective sample and represented as the averaged log2 normalized fold increase above donor- and time point-matched uninfected cells. Error bars represent the mean +/- SD. (TIF) [file ppat.1006164.s005.tif]

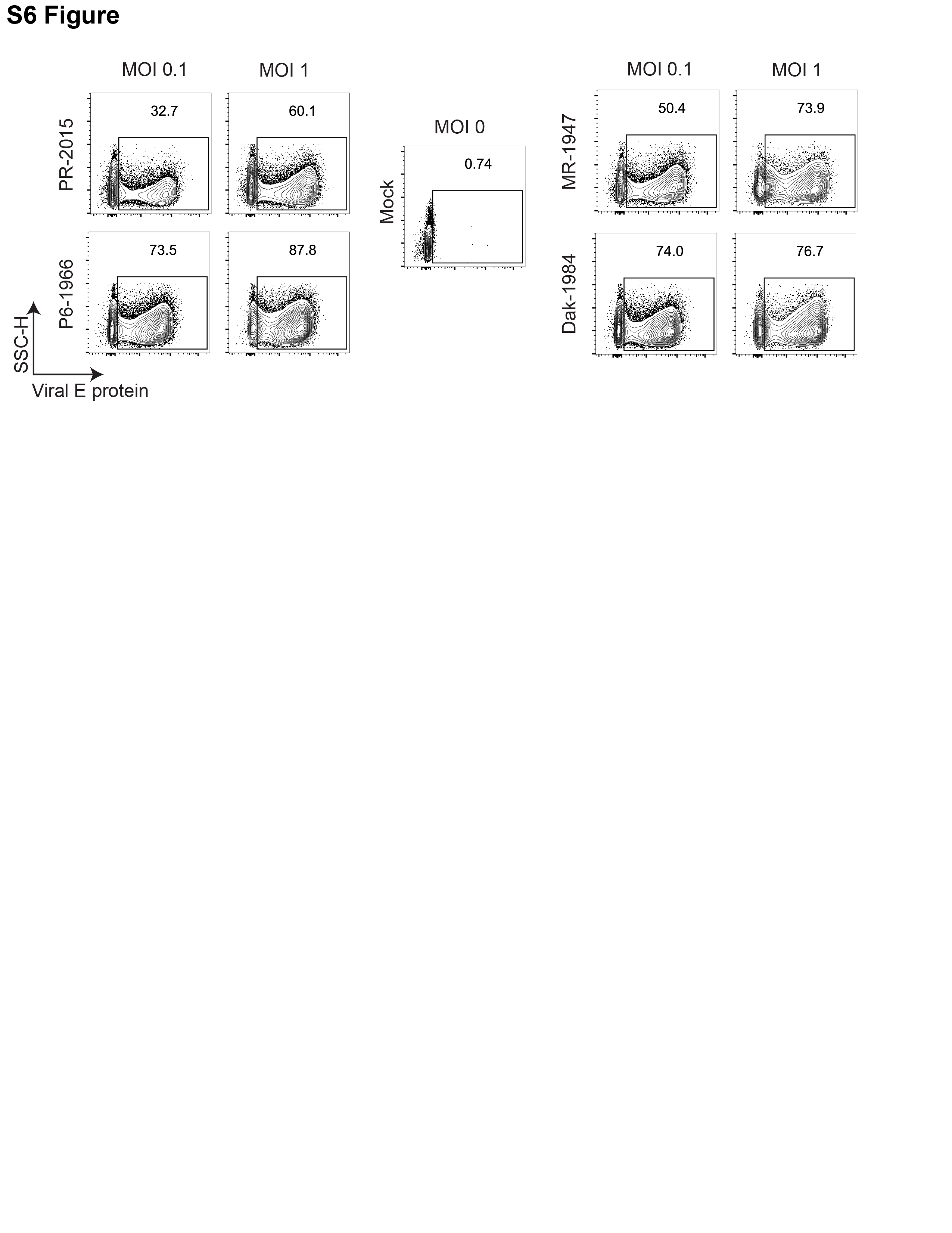

Supplement: S6 Fig — Representative flow plots of A549 cells infected with indicated ZIKV strain at MOI of 0.1 or 1 for 48hrs and labeled for the presence of viral E protein. Data is representative of two independent experiments. (TIF) [file ppat.1006164.s006.tif]
