# Supplementary material for: Zika Virus Antagonizes Type I Interferon Responses during Infection of Human Dendritic Cells
Source: PLoS Pathog. 2017 Feb 2;13(2):e1006164. doi: 10.1371/journal.ppat.1006164 (PMC5289613; doi:10.1371/journal.ppat.1006164)
Supplement: S1 Table — Information about the ZIKV strains used throughout these studies, nucleotide similarity between coding regions of ZIKV strain genomes, and amino acid differences between viral proteins of ZIKV strains. CDS- coding DNA sequence, V- Vero cell, SM- suckling mouse brain, Ap61- Aedes pseudoscutellaris cell line, C6- Aedes albopictus clone C6/36 cell line. (PDF) [file ppat.1006164.s007.pdf]

S1 Table

Zika virus strain information

	PR-2015	P6-1966	MR-1947	Dak-1984
**Strain**	PRVABC59	P6-740	MR766	DakAr 41524
**Accession**	KX601168	KX601167	KX601169	KX601166
**Lineage**	Asian	Asian	East African	West African
**Country**	Puerto Rico	Malaysia	Uganda	Senegal
**Date**	2015	1966	1947	1984
**Passages**	V(4)	SM(6), V(3)	SM(149), V(3)	Ap61(1), C6(1), V(1)

Nucleotide similarity of the CDS between strains

	PR-2015	P6-1966	MR-1947	Dak-1984
**PR-2015**	100	95.5	88.6	88.6
**P6-1966**	95.5	100	89.9	89.9
**MR-1947**	88.6	89.9	100	93.4
**Dak-1984**	88.6	89.9	93.4	100

Amino acid changes as compared to PR-2015

		P6-1966		MR-1947		Dak-1984	
**Protein**	**Total AAs**	**# of changes**	**% changes**	**# of changes**	**% changes**	**# of changes**	**% changes**
**ancC**	**122**	1	0.8%	6	4.9%	5	4.1%
**C**	**104**	1	1.0%	4	3.8%	4	3.8%
**anc**	**18**	0	0.0%	2	11.1%	1	5.6%
**preM**	**168**	2	1.2%	10	6.0%	10	6.0%
**pr**	**93**	2	2.2%	7	7.5%	7	7.5%
**M**	**75**	0	0.0%	3	4.0%	3	4.0%
**E**	**504**	6	1.2%	19	3.8%	11	2.2%
**NS1**	**352**	2	0.6%	9	2.6%	7	2.0%
**NS2A**	**226**	2	0.9%	9	4.0%	10	4.4%
**NS2B**	**130**	0	0.0%	2	1.5%	2	1.5%
**NS3**	**617**	3	0.5%	10	1.6%	13	2.1%
**NS4A**	**127**	0	0.0%	1	0.8%	2	1.6%
**2K**	**23**	0	0.0%	0	0.0%	0	0.0%
**NS4B**	**251**	7	2.8%	10	4.0%	9	3.6%
**NS5**	**903**	16	1.8%	35	3.9%	35	3.9%
**Structural**	**794**	9	1.1%	35	4.4%	26	3.3%
**Non-structural**	**2629**	30	1.1%	76	2.9%	78	3.0%
**Polyprotein**	**3423**	39	1.1%	111	3.2%	104	3.0%
