# Supplementary material for: Zika Virus Antagonizes Type I Interferon Responses during Infection of Human Dendritic Cells
Source: PLoS Pathog. 2017 Feb 2;13(2):e1006164. doi: 10.1371/journal.ppat.1006164 (PMC5289613; doi:10.1371/journal.ppat.1006164)
Supplement: S2 Table — moDCs were left untreated (“Mock”), transfected with RIG-I agonist (10ng/1e5 cells), or infected with ZIKV PR-2015, P6-1966, MR-1947, or Dak-1984 at MOI of 1 (n = 7 donors). Cytokine levels in the supernatants were determined by multiplex bead array at 24hrs post-agonist transfection or 48hrs post-infection. All values are represented in “pg/mL”. Cytokine levels that were below the lower limit of detection are indicated as not detected or “ND”. LLOQ, lower limit of quantitation. (PDF) [file ppat.1006164.s008.pdf]

S2 Table

	moDCs												
	**LLOQ**	**Mock**		**RIG-I Agonist**		**PR-2015**		**P6-1966**		**MR-1947**		**Dak-1984**	
		**Mean**	**SD**	**Mean**	**SD**	**Mean**	**SD**	**Mean**	**SD**	**Mean**	**SD**	**Mean**	**SD**
**IL-1β**	5	ND	ND	ND	ND	ND	ND	ND	ND	ND	ND	ND	ND
**IL-10**	1	ND	ND	11.1	6.7	ND	ND	7.1	5.8	6.5	6.9	5.4	5.9
**IL-13**	0.5	3.2	0.7	2.5	0.7	3.5	0.6	1.7	0.4	1.9	0.3	1.5	0.4
**IL-6**	0.5	16.3	10.8	263.3	162.6	24.8	12.0	142.5	69.3	51.4	44.7	47.8	40.9
**IL-12**	5	11.1	12.2	45.9	6.8	32.2	69.0	50.8	60.7	27.7	31.4	24.0	25.3
**RANTES**	10	8.7	1.0	338.8	148.2	8.9	1.0	10.4	4.1	8.8	1.2	8.6	1.2
**Eotaxin**	0.5	ND	ND	ND	ND	ND	ND	ND	ND	ND	ND	ND	ND
**IL-17**	5	ND	ND	ND	ND	ND	ND	ND	ND	ND	ND	ND	ND
**MIP-1α**	5	215.4	273.0	4505.2	1515.9	156.4	178.4	230.8	191.1	231.4	283.5	211.5	250.2
**GM-CSF**	0.5	ND	ND	1145.0	692.9	ND	ND	ND	ND	ND	ND	ND	ND
**MIP-1β**	5	227.2	136.1	3776.5	2074.6	158.6	68.2	164.1	84.4	161.5	79.7	150.3	75.8
**MCP-1**	5	1541.7	1153.6	9969.7	2321.0	2004.9	1688.8	4044.3	3278.8	2944.3	2552.6	3112.2	2872.8
**IL-15**	40	ND	ND	89.4	15.3	ND	ND	ND	ND	ND	ND	ND	ND
**IL-5**	5	ND	ND	ND	ND	ND	ND	ND	ND	ND	ND	ND	ND
**IFNγ**	1	3.5	1.7	9.3	2.7	3.2	1.4	5.6	1.6	5.7	2.8	5.3	2.8
**IL-1RA**	15	4988.8	1702.6	6498.4	2015.5	4952.7	862.0	8166.8	1551.7	7774.8	883.4	7571.7	1193.6
**TNFα**	0.5	ND	ND	49.8	36.7	ND	ND	ND	ND	ND	ND	ND	ND
**IL-2**	1	ND	ND	ND	ND	ND	ND	ND	ND	ND	ND	ND	ND
**IL-7**	10	27.4	5.0	52.2	9.1	25.9	6.2	20.2	6.1	17.4	9.3	14.5	5.7
**IP-10**	0.5	4.3	1.0	1257.5	381.9	10.0	10.1	108.8	91.5	112.8	109.4	101.5	100.3
**IL-2R**	20	15.6	8.7	77.1	8.0	14.8	8.0	16.2	6.6	18.4	9.4	13.6	10.1
**MIG**	5	ND	ND	27.2	10.2	13.0	25.5	27.7	56.9	30.1	70.2	31.0	69.5
**IL-4**	1	ND	ND	1689.3	923.3	ND	ND	ND	ND	ND	ND	ND	ND
**IL-8**	5	1003.6	1019.8	2183.9	1754.0	1188.1	1086.5	3057.1	2267.7	1915.4	1251.7	1891.9	1312.2
**IFNα**	5	25.5	11.8	153.0	28.3	28.7	14.6	44.7	16.9	46.1	33.5	46.6	29.6
**IFNβ**	11	ND	ND	36.7	9.2	ND	ND	ND	ND	1.8	4.8	ND	ND
**IL-29**	57	56.9	8.6	618.3	84.3	64.9	7.5	76.4	13.3	106.1	46.3	76.4	13.3
