# Supplementary material for: Zika Virus Antagonizes Type I Interferon Responses during Infection of Human Dendritic Cells
Source: PLoS Pathog. 2017 Feb 2;13(2):e1006164. doi: 10.1371/journal.ppat.1006164 (PMC5289613; doi:10.1371/journal.ppat.1006164)
Supplement: S3 Table — Monocytes were left untreated (“Mock”), treated with LPS (100ng/mL), or infected with ZIKV PR-2015 at MOI of 1 (n = 4–5 donors). Cytokine levels in the supernatants were determined by multiplex bead array 24hrs later. Cytokines that were not assayed are indicated as “-“. (PDF) [file ppat.1006164.s009.pdf]

S3 Table

			Monocytes					
			Mock		LPS		PR-2015	
	**Limit of detection (pg/ml)**	**Unit of expression**	**Mean**	**SD**	**Mean**	**SD**	**Mean**	**SD**
**IL-1b**	7.2	pg/ml	100.5	76.4	2372.7	1367.5	49.9	21.9
**IL-6**	2.5	ng/ml	2.3	0.6	23.1	6.3	2.7	0.8
**IL-10**	3.3	pg/ml	29.1	20.0	2077.7	1172.2	26.3	20.4
**IL-12p70**	1.9	pg/ml	1.5	0.7	33.2	55.9	1.3	2.0
**TNF**	3.7	pg/ml	12.0	8.3	2227.1	1416.5	15.5	12.4
**IFN-a**	1.5	pg/ml	-	-	-	-	-	-
**MCP-1**	2.7	ng/ml	26.3	16.1	23.8	12.6	33.5	12.2
**Rantes**	1.0	pg/ml	57.6	63.9	399.2	299.7	51.9	47.8
**IL-8**	0.2	ng/ml	67.3	44.1	172.4	27.0	81.4	37.4
**MIG-1**	2.5	pg/ml	133.2	156.2	473.8	409.8	591.9	681.0
**IP-10**	2.8	pg/ml	58.7	32.5	22.1	40.7	21.0	10.7
