# Supplementary material for: Zika Virus Antagonizes Type I Interferon Responses during Infection of Human Dendritic Cells
Source: PLoS Pathog. 2017 Feb 2;13(2):e1006164. doi: 10.1371/journal.ppat.1006164 (PMC5289613; doi:10.1371/journal.ppat.1006164)
Supplement: S4 Table — mDCs were left untreated (“Mock”), treated with LPS (100ng/mL), or infected with ZIKV PR-2015 at MOI of 1 (n = 4–5 donors). Cytokine levels in the supernatants were determined by multiplex bead array 24hrs later. Cytokines that were not assayed are indicated as “-“. (PDF) [file ppat.1006164.s010.pdf]

S4 Table

			Myeloid DCs					
			Mock		LPS		PR-2015	
	Limit of detection (pg/ml)	Unit of expression	Mean	SD	Mean	SD	Mean	SD
**IL-1b**	7.2	pg/ml	5.0	5.1	19.8	5.3	10.5	9.5
**IL-6**	2.5	ng/ml	0.0	0.0	1.4	1.1	0.2	0.3
**IL-10**	3.3	pg/ml	1.9	2.5	20.5	10.6	6.0	12.0
**IL-12p70**	1.9	pg/ml	1.9	2.0	15.7	9.2	2.2	0.9
**TNF**	3.7	pg/ml	9.7	4.8	62.4	28.9	19.6	6.8
**IFN-a**	1.5	pg/ml	-	-	-	-	-	-
**MCP-1**	2.7	ng/ml	6.0	4.8	6.7	3.5	17.6	16.0
**Rantes**	1.0	pg/ml	7.6	4.1	108.8	103.7	17.1	13.9
**IL-8**	0.2	ng/ml	3.3	1.8	13.4	6.8	7.3	2.9
**MIG-1**	2.5	pg/ml	77.7	99.8	361.9	368.1	369.2	470.4
**IP-10**	2.8	pg/ml	4.8	4.4	32.9	22.9	78.1	58.0
