# Supplementary material for: Zika Virus Antagonizes Type I Interferon Responses during Infection of Human Dendritic Cells
Source: PLoS Pathog. 2017 Feb 2;13(2):e1006164. doi: 10.1371/journal.ppat.1006164 (PMC5289613; doi:10.1371/journal.ppat.1006164)
Supplement: S5 Table — pDCs were left untreated (“Mock”), treated with R848 (1μg/mL) or infected with ZIKV PR-2015 at MOI of 1 (n = 4–5 donors). Cytokine levels in the supernatants were determined by multiplex bead array 24hrs later. (PDF) [file ppat.1006164.s011.pdf]

S5 Table

			Plasmacytoid DCs					
			Mock		R848		PR-2015	
	Limit of detection (pg/ml)	Unit of expression	Mean	SD	Mean	SD	Mean	SD
**IL-1b**	7.2	pg/ml	4.7	5.9	3.0	1.7	24.6	25.6
**IL-6**	2.5	ng/ml	4.2	4.1	271.6	141.1	273.3	351.1
**IL-10**	3.3	pg/ml	3.4	5.2	0.2	0.3	16.6	7.6
**IL-12p70**	1.9	pg/ml	2.8	2.9	5.0	3.4	6.3	6.6
**TNF**	3.7	pg/ml	31.2	19.0	1087.4	885.7	53.2	38.8
**IFN-a**	1.5	pg/ml	6.9	2.8	13059.4	8911.5	76.6	59.6
**MCP-1**	2.7	ng/ml	3.2	2.8	9.5	7.2	28.8	30.4
**Rantes**	1.0	pg/ml	3.3	3.3	260.9	170.6	7.9	3.9
**IL-8**	0.2	ng/ml	0.3	0.3	2.3	1.7	0.4	0.2
**MIG-1**	2.5	pg/ml	103.2	82.5	1479.1	1977.1	238.3	298.8
**IP-10**	2.8	pg/ml	4.1	3.2	1136.1	877.8	51.2	55.3
